# Cross-talk between miR-471-5p and autophagy component proteins regulates LC3-associated phagocytosis (LAP) of apoptotic germ cells

**DOI:** 10.1038/s41467-017-00590-9

**Published:** 2017-09-19

**Authors:** Subbarayalu Panneerdoss, Suryavathi Viswanadhapalli, Nourhan Abdelfattah, Benjamin C. Onyeagucha, Santosh Timilsina, Tabrez A. Mohammad, Yidong Chen, Michael Drake, Kristiina Vuori, T. Rajendra Kumar, Manjeet K. Rao

**Affiliations:** 1 Department of Cell Systems and Anatomy, University of Texas Health Science Center at San Antonio, 7703 Floyd Curl Drive, San Antonio, Texas 78229 USA; 2 Greehey Children’s Cancer Research Institute 8403 Floyd Curl Drive, University of Texas Health Science Center at San Antonio, San Antonio, Texas 78229 USA; 3 Department of Obstetrics and Gynecology, University of Texas Health Science Center at San Antonio, 7703 Floyd Curl Drive, San Antonio, Texas 78229 USA; 4 Department of Laboratory Animal Resources, University of Texas Health Science Center at San Antonio, 7703 Floyd Curl Drive, San Antonio, Texas 78229 USA; 5 Sanford-Burnham Medical Research Institute, 10901 North Torrey Pines Road, La Jolla, California 92037 USA; 6 Department of Obstetrics and Gynecology, University of Colorado Denver, 12700 E. 19th Avenue, Aurora, Colorado 80045 USA

## Abstract

Phagocytic clearance of apoptotic germ cells by Sertoli cells is vital for germ cell development and differentiation. Here, using a tissue-specific miRNA transgenic mouse model, we show that interaction between *miR-471-5p* and autophagy member proteins regulates clearance of apoptotic germ cells via LC3-associated phagocytosis (LAP). Transgenic mice expressing *miR-471-5*p in Sertoli cells show increased germ cell apoptosis and compromised male fertility. Those effects are due to defective engulfment and impaired LAP-mediated clearance of apoptotic germ cells as *miR-471-5p* transgenic mice show lower levels of Dock180, LC3, Atg12, Becn1, Rab5 and Rubicon in Sertoli cells. Our results reveal that Dock180 interacts with autophagy member proteins to constitute a functional LC3-dependent phagocytic complex. We find that androgen regulates Sertoli cell phagocytosis by controlling expression of *miR-471-5p* and its target proteins. These findings suggest that recruitment of autophagy machinery is essential for efficient clearance of apoptotic germ cells by Sertoli cells using LAP.

## Abstract (web summary)

Although phagocytic clearance of apoptotic germ cells by Sertoli cells is essential for spermatogenesis, little of the mechanism is known. Here the authors show that Sertoli cells employ LC3-associated phagocytosis (LAP) by recruiting autophagy member proteins to clear apoptotic germ cells.

## Introduction

Phagocytosis is an evolutionarily conserved cellular event that plays a vital role in maintaining tissue homeostasis by clearing apoptotic cells during several developmental processes throughout life. In addition to conventional phagocytosis, LC3-associated phagocytosis (LAP) is reported to play an equally important role in the clearance of phagocytosed dead cells by macrophages^1^. LAP engages several members of autophagy pathway that facilitate recruitment of LC3 to single-membrane phagosomes, resulting in prompt phagosome maturation and degradation of dead cells. The phagocytosis is particularly important during spermatogenesis, when more than half of developing male germ cells undergo apoptosis and are cleared by Sertoli nurse cells^2^. Though LAP has not been investigated in the Sertoli cells, the rapid and efficient degradation of apoptotic germ cells by Sertoli cells is presumed to be crucial for proper germ cell development and differentiation. Little was known about the molecular mechanism that regulates Sertoli cell phagocytosis until recently when it was shown that cytoplasmic engulfment protein Elmo1, which promotes internalization of dying cells, plays an essential role in Sertoli cell phagocytosis^3^. Elmo1-knockout mice had increased germ cell apoptosis, uncleared apoptotic germ cells, and defective germ cell development, resulting in reduced germ cell output^3^. The uncleared apoptotic germ cells were due to Sertoli cells’ impaired ability to efficiently engulf apoptotic germ cells^3^. Though insightful, much need still remains to understand the detailed mechanisms that regulate discrete steps of the phagocytic process in Sertoli cells and also whether Sertoli cells employ LAP for efficient clearance of germ cells.

In this study, by generating a novel Sertoli cell-specific microRNA (miRNA) transgenic mice, we report that *miR-471-5p* plays an important role in regulating LAP in Sertoli cells. Increased expression of *miR-471-5p* inhibited germ cell engulfment as well as LAP-mediated germ cell clearance in Sertoli cells. The impaired engulfment and clearance of apoptotic germ cells is largely because of the altered levels and activity of several phagocytosis/autophagy-associated proteins, including Dock180 (dedicator of cytokinesis 1), LC3, Atg12 (autophagy related 12), Becn1 (beclin1, autophagy related) Tecpr1 (tectonin β-propeller repeat-containing protein 1) and rubicon (RUN-domain protein as Beclin 1 interacting and cysteine-rich containing). Dock180 is a guanine nucleotide exchange factor that along with cytoplasmic engulfment protein Elmo1 induces Rac1-GTPase and thereby promotes engulfment^3^. The Dock180–Elmo1–Rac1 signaling network plays a vital role in Sertoli cell phagocytosis^3^. LC3 is an autophagy protein, lapidated form (LC3II) of which is recruited to the double-membrane autophagosome and also to the single-membrane phagosome during LAP^4^. Atg12 is a key autophagosomal protein that interacts with Atg5 and Atg16L complex to play a role in autophagy as well as in LAP^5^. Rubicon is a PI3K-associated protein reported to be essential for initiating LAP^5^. Becn1 is an autophagy protein, which plays a critical role in the maturation of LC3-containing phagosomes by facilitating the recruitment of Rab5 GTPase, leading to acidification of dead cell containing LC3-decorated phagosomes^5, 6^. Tecpr1 is a component of the autophagy network that interacts with the Atg12–Atg5 complex to regulate fusion between autophagosomes and lysosomes^4, 7^. Though it is unclear whether or not Tecpr1 is directly involved in the LC3 recruitment to the phagosome, however, it is known that Tecpr1 function requires PI3K activity, which is vital for LAP^4, 8^. Importantly, we show that Dock180, in addition to engulfment, plays an equally vital role in clearance of apoptotic germ cells by directly interacting with LC3 and other autophagy component proteins in mammalian cells in general and Sertoli cells in particular. Furthermore, we show that androgen plays a crucial role in clearance of apoptotic germ cells by controlling the expression of *miR-471-5p* and its target autophagy-associated proteins in the Sertoli cells. Our results showing abundant expression of Dock180 and autophagy-associated proteins in the Sertoli cells and their involvement in regulating LAP suggest that convergence of both autophagy and phagocytosis pathways is essential for Sertoli cells to efficiently degrade and clear apoptotic germ cells.

## Results

### *MiR-471-5p* is important for complete fertility

**Fig. 1:**
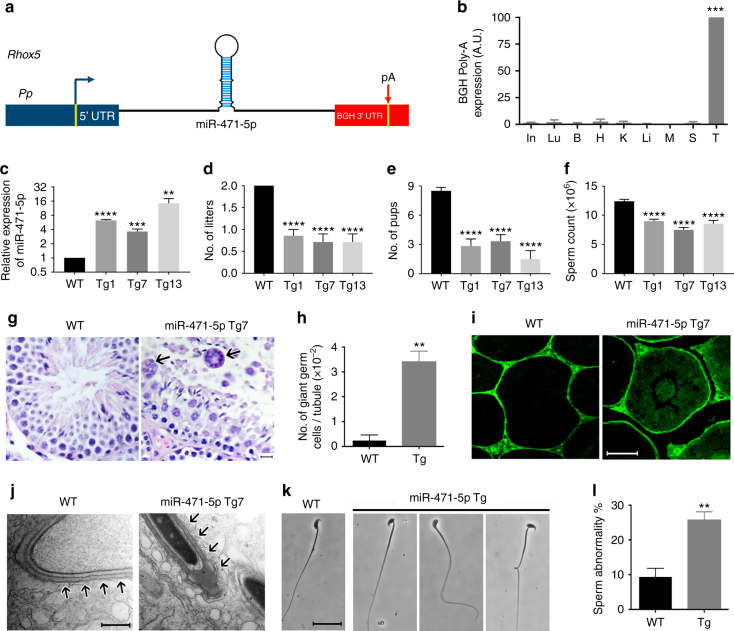
Reproductive defects in miR-471-5p transgenic (Tg) mice. **a** Schematic of the construct used to generate miR-471-5p transgenic mice (miR-471-5pTg). *Pp* indicates 0.6 kb proximal promoter from Rhox5 gene. *MiR-471* represents primary sequence of miR-471-5p. pA represents bovine growth hormone polyA sequence. **b** qRT-PCR analyses of RNA isolated from the vital organs of miR-471-5p Tg using BGH polyA primers. ***p = 0.0002, one-way ANOVA followed by Dunnett’s multiple comparisons test. **c** qRT-PCR was used to quantitate the levels of mature *miR-471-5p* in total testicular RNA from three transgenic lines (Tg1, Tg7, and Tg13) and normal control mice (wild-type; *WT*). Bar graph represents average fold increase of *miR-471-5p* expression over endogenous *miR-471-5p* levels. Expression normalized to RNU19 or 5S; ****p < 0.0001,***p = 0.001, **p = 0.0036; two-tailed unpaired student *t*-test. **d**, **e** Total number of litters (**d**) and pups per litter (**e**) obtained from 2 months mating between control male and control female (WT) and between miR-471-5pTg male (from Tg1, 7 and 13 lines) with control female littermates (*n* = 10 for WT; and *n* = 10 for each of the three miR-471-5p transgenic lines). ****p < 0.0001; one-way ANOVA followed by Dunnett’s multiple comparisons test. **f** Caudal sperm count in miR-471-5p Tg and control (WT) mice (*n* = 7 for WT and *n* = 7 for miR-471-5pTg mice), ****p < 0.0001; one-way ANOVA followed by Dunnett’s multiple comparisons test. **g** Hematoxylin and eosin-stained sections of testes of control (WT) and miR-471-5pTg mice (line Tg7). *Arrows* indicate multinucleated giant germ cells in miR-471-5pTg mice testis. **h** Average number of giant cells per tubule in testes of control (WT) and miR-471-5pTg mice, **p = 0.0023, two-tailed unpaired student *t*-test. **i** Appearance of biotin tracer dye in the adluminal compartment of some seminiferous tubules of miR-471-5pTg mice suggests a leaky blood–testis barrier. Wild-type (*WT*) control mice show restriction of biotin tracer to the basal compartment. **j** Ultrastructural defects in miR-471-5pTg (line Tg7) mice testis. Transmission electron microscopy of testicular section from control mouse shows normal morphology. The acrosome extends over the apex and over the dorsal curvature of the head. Apical ectoplasmic specialization is well recognized and is seen over the entire acrosome region. Testicular sections from miR-471-5pTg mice show disruption and loss of apical ES (*arrows*). **k** Phase-contrast microscopy of sperm shows abnormal head/tail morphology in miR-471-5pTg mice. **l** Histogram shows average number of abnormal spermatozoa in miR-471-5p Tg (average of Tg1, Tg7, and Tg13 lines, 3 animals/line) and normal control (WT) mice; **p = 0.0044, two-tailed unpaired student *t*-test. *Scale bar* indicates 50 μm **g**, **i**), 500 nm (**j**), 25 μm (**k**). ANOVA: analysis of variance. In = Intestine; Lu = Lungs; B = Brain; H = Heart; K = Kidney; Li = Liver; M = Muscle; S = Spleen; T = Testis

We recently showed that several miRNAs are highly expressed in Sertoli nurse cells in an androgen-dependent manner^9^. Of those miRNAs, we find that *miR-471-5p* expression begins to peak at day 12 in Sertoli cells^9^, coincident with the initiation of androgen-dependent spermatogenic events in the mouse testis. To address the function of *miR-471-5p* in Sertoli cells, we generated *miR-471-5p* transgenic mice (miR-471-5p Tg) by inserting *miR-471-5p* mature sequences flanked by ~ 250 base pairs (bp) of the endogenous locus driven by a well-established Sertoli cell-specific *Rhox5* proximal promoter (*Rhox5 Pp*) (Fig. 1a), which drives transgene expression in the Sertoli cells at all stages of the seminiferous epithelial cycle^10, 11^. We generated 10 independent transgenic mouse lines containing the *miR-471-5p-BGH polyA* transgene (Supplementary Fig. 1a), all of which expressed the transgene in their testes (Fig. 1b and Supplementary Fig. 1b). Real-time quantitative PCR (RT-qPCR) analysis in the control (wild type) and miR-471-5pTg mice showed that *miR-471-5p* levels ranged from ~ 2–4-fold to endogenous levels to ~ 8–16-fold more than that of the endogenous levels (Fig. 1c).

Next, we assessed whether *miR-471-5p* expression in Sertoli cells resulted in any reproductive defects. Fertility analyses were carried out in all 10 transgenic lines, while the rest of the experiments were performed in three transgenic lines (Tg1, 7, and 13) that showed various levels of transgene expression. Analysis of miR-471-5pTg mice revealed several reproductive defects leading to compromised male fertility. In comparison with wild-type controls, miR-471-5pTg mice from all 10 *miR-471-5p* transgenic lines, including Tg1, 7 and 13 not only sired significantly fewer litters but each litter also had fewer pups (Fig. 1d, e and Supplementary Fig. 1c, d). Furthermore, mice from Tg1, 7 and 13 exhibited significantly lower caudal epididymal sperm counts (Fig. 1f). The compromised fertility in miR-471-5pTg male mice is due to testicular defects as histological analysis of testes sections showed more multinucleated giant cells in the miR-471-5pTg mice (Fig. 1g, h), indicating that spermatocytes did not undergo complete meiosis^3^. Multinucleated giant cells are often noticed in testes when spermatogenesis is impaired in mice and in infertile men^12, 13^. The premeiotic germ cell developmental defect could be due to disrupted cellular organization of the seminiferous tubules, as we observed germ cell sloughing in many tubules (Supplementary Fig. 2a), a phenotype consistent with impaired cell-cell adhesion of Sertoli cells at the blood–testis barrier (BTB). To further substantiate these findings, we determined the functional integrity of the BTB of miR-471-5pTg mice by injecting EZ-link sulfo-NHS-LC-biotin tracer, which cannot penetrate the intact BTB. Histological analysis of testicular sections showed that dye penetrated into the luminal side of some tubules in the miR-471-5pTg mice, suggesting disrupted integrity of the BTB, whereas distribution of the dye was, as expected, restricted only to the basement side in control mice (Fig. 1i and Supplementary Fig. 2b). To further test BTB functional integrity, we determined expression of tight-junction proteins in testes of miR-471-5pTg mice. RT-qPCR analysis showed significantly lower and disrupted expression pattern of *occludin* (*Ocln*) and *claudin 3* (*cldn3*) in miR-471-5pTg mice compared with those in control mice, indicating impaired BTB integrity (Supplementary Fig. 2c, d). In addition, we observed significantly lower levels of Sertoli cell-specific gene desmocollin 2 (*Dsc2*), which is an integral component of desmosomes^14^ and is known to play an important role in Sertoli cell-germ cell adhesion^15^, in miR-471-5pTg mice (Supplementary Fig. 2e). Those functional data further suggest that *miR-471-5p* plays an important role in regulating germ cell attachment to Sertoli cell as well as BTB integrity, a well-established androgen-dependent event^16^. Since pre- and postmeiotic germ cell maturation takes place at the opposite ends of Sertoli cells, we tested whether *miR-471-5p* expression in Sertoli cells is also important for postmeiotic germ cell differentiation. Ultrastructural analyses by electron microscopy showed that *miR-471-5p* overexpression in Sertoli cells disrupts adherens junctions between Sertoli cells and elongating spermatids (apical ectoplasmic specialization; ES) compared with intact apical ES in seminiferous tubules in wild-type mice (Fig. 1j). That finding is significant as apical ES is presumed to play vital roles in the maturation of differentiating germ cells by controlling orientation, positioning, and head morphology of the spermatid^17^. In addition, sperm from miR-471-5pTg mice exhibited various morphological abnormalities. The most striking sperm defects included round heads (globozoospermia), bent heads, and kinked tails (Fig. 1k, l).

**Fig. 2:**
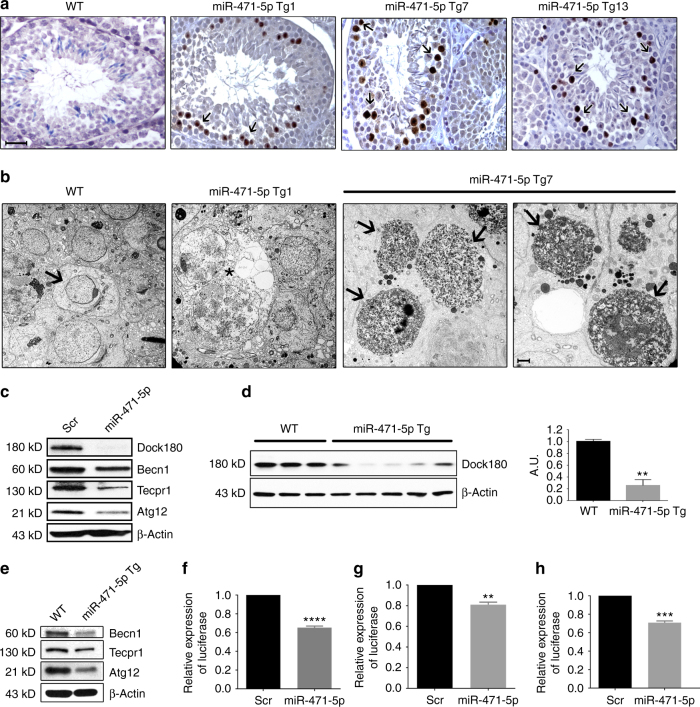
*MiR-471-5p* regulates germ cell apoptosis/necrosis and targets autophagy-associated proteins in Sertoli cells. **a** TUNEL analysis of testes sections from three miR-471-5p transgenic lines (Tg1, Tg7, and Tg13) showed significantly increased number of apoptotic/necrotic germ cells. **b** Electron microscopic observation of miR-471-5pTg mice testis showed drastically increased germ cell apoptosis/necrosis in two independent lines Tg1 and Tg7. *Asterisks* (*) indicate presence of large vacuoles in miR-471-5pTg mice. *Black arrows* in miR-471-5p Tg7 indicate apoptotic germ cells. WT testis showed normal acrosome and spermatogenesis. **c** Western blot analysis on 15p1 Sertoli cells transfected with scramble (*Scr*) or *miR-471-5p* mimic (*miR-471-5p*) using antibodies against Dock180 (1:1000), Becn1 (1:1000), Tecpr1 (1:1000), and Atg12 (1:1000). Gel photograph is representative of three independent experiments. Bar graphs showing quantification of band intensities are shown in Supplementary Fig. 3b. **d** Western blot analysis on total testis from WT and miR-471-5pTg using antibody against Dock180. β-Actin (1:50,000) was used as a loading control. Gel photograph is representative of three independent experiments (WT: *n* = 3 mice per experiment; miR-471-5pTg: 5 mice per experiment). Band intensities were quantified using ImageJ software, **p = 0.0016, two-tailed unpaired student *t*-test. **e** Western blot analysis on purified Sertoli cells isolated from WT and miR-471-5p Tg mice using antibodies against indicated proteins. Gel photograph is representative of three independent experiments. Purified Sertoli cells were pooled from six each of WT and miR-471-5p Tg mice/experiment. Quantification of band intensities are shown in Supplementary Fig. 3e. **f**–**h** TM4 Sertoli cells were cotransfected with scramble or *miR-471-5p* mimic and Renilla luciferase expression construct pRL–CMV as well as firefly luciferase construct containing *Dock180* (**f**), *ATG12* (**g**), or *Becn1* (**h**) 3′-UTR. Firefly luciferase activity for each sample was normalized with Renilla luciferase activity. Graph shows mean ± SEM of three independent experiments (performed in duplicate for each experiments). ****p < 0.0001; **p = 0.0012; *p* = 0.0001, two-tailed unpaired student *t*-test. *Scale bar* indicates 25 μm (**a**), 2 μm (**b**)

We next assessed whether reduced sperm output in miR-471-5pTg mice was due to increased germ cell death. Terminal deoxynucleotidyl transferase dUTP nick-end labeling (TUNEL) assay and immunohistochemical analyses using antibody against cleaved caspase 3 on testicular sections revealed that miR-471-5pTg mice had significantly more apoptotic/necrotic germ cells (number of positive cells/tubules) than littermate control mice (Fig. 2a, Supplementary Figs. 2f and 3a). Ultrastructural analyses on miR-471-5pTg mice testes further confirmed these findings (Fig. 2b). Many seminiferous tubules had more than 20 apoptotic/necrotic germ cells per tubule, suggesting an increased incidence and frequency of uncleared apoptotic/necrotic germ cells in miR-471-5pTg mice.

### *MiR-471-5p* regulates phagocytosis/autophagy proteins

To address the underlying mechanism of reproductive defects including uncleared apoptotic germ cells in miR-471-5pTg mice, we identified target gene networks of *miR-471-5p* by performing Ago2-RNA immunoprecipitation followed by deep sequencing. Putative mRNA targets of *miR-471-5p* were identified by comparing the Ago2 IP/deep sequencing profiles *of miR-471-5p-*transfected Sertoli cells with those of scramble-transfected Sertoli cells. The mRNAs whose association with Ago2 increased upon *miR-471-5p* overexpression were much more likely to contain specific *miR-471-5p* seed matches and to have their overall mRNA levels decrease in response to the miRNA overexpression than expected by chance. Many of these genes including *Dock180*, *Atg12*, *Becn1* and *Tecpr1* are associated with phagocytosis/LAP (Supplementary Table 1). To address whether these genes are indeed targets of *miR-471-5p* and are associated with Sertoli cell phagocytosis/LAP, we first determined their protein levels in *miR-471-5p* mimic transfected cells. Western blot analyses revealed that overexpression of *miR-471-5p* mimic in 15P1 and TM4 Sertoli cells reduced expression of Dock180, Tecpr1, Atg12, and Becn1 (Fig. 2c and Supplementary Fig. 3b–d). Supporting the in vitro findings, we observed drastically reduced levels of Dock180, Tecpr1, Atg12, and Becn1 proteins in the testis and purified Sertoli cells of miR-471-5pTg mice in comparison with wild-type sibling controls (Fig. 2d, e and Supplementary Fig. 3e). Moreover, our bioinformatics analysis predicted that the 3′-untranslated region (UTR) and coding regions of these genes contain binding sites for *miR-471-5p* (Supplementary Fig. 3f and data not shown). To determine whether *miR-471-5p* regulates target gene expression by binding to their putative sites in these genes, we performed luciferase assay. Co-transfection of 3′-UTR-luciferase constructs of *Dock180*, *Atg12*, *Becn1 and miR-471-5p* mimic or scramble in Sertoli cell lines showed reduced levels of luciferase activity, indicating that *miR-471-5p* targets *Dock180*, *Atg12*, and *Becn1* levels by binding to their 3′-UTR region (Fig. 2f–h). Taken together, these findings suggest that autophagy-associated proteins are highly expressed in Sertoli cells and *miR-471-5p* regulates both engulfment and clearance of apoptotic germ cells by targeting those proteins.

### *MiR-471-5p* regulates apoptotic germ cell engulfment

**Fig. 3:**
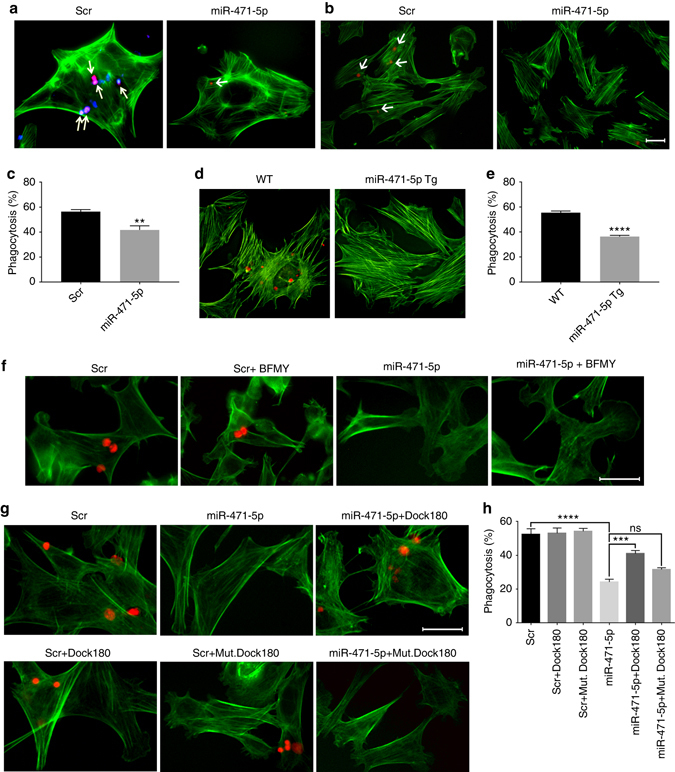
*MiR-471-5p* regulates Sertoli cell phagocytosis of apoptotic germ cells. **a** Immunofluorescence analysis showing phagocytosis of apoptotic germ cells by primary Sertoli cells transfected with scramble or *miR-471-5p* mimic. Scramble and *miR-471-5p* mimic-transfected Sertoli cells were fed with apoptotic male germ cells labeled with pHrodo. *Arrows* indicate engulfed germ cells. To show cell morphology, Sertoli cells were stained with FITC-labeled actin (*green*). **b** Immunofluorescence analysis showing phagocytosis of apoptotic germ cells by 15P1 Sertoli cells transfected with scramble or *miR-471-5p* mimic (miR-471-5p). Scramble (*Scr*) and *miR-471-5p mimic*-transfected 15P1 Sertoli cells were fed with apoptotic mouse germ cells labeled with pHrodo (*red*). *Arrows* indicate Sertoli cells with engulfed germ cells. **c** Histogram showing percentage of control and *miR-471-5p* mimic-transfected Sertoli cells engulfing apoptotic germ cells as derived from the flow cytometric analysis. The data are presented as mean ± SEM of three independent experiments; **p = 0.001; two-tailed unpaired student *t*-test. **d** In vitro phagocytosis assay in primary Sertoli cells purified from wild-type (*WT*) or miR-471-5pTg. Phagocytosis assay was performed as described in **a**. **e** Histogram showing percentage of Sertoli cells engulfing apoptotic germ cells as derived from immunofluorescence analysis. The data are presented as mean ± SEM of three independent experiments. Primary Sertoli cells were pooled from 5 miR-471-5p Tg mice/experiment. ****p < 0.0001, two-tailed unpaired student *t*-test. **f** Immunofluorescence analysis showing phagocytosis of pHrodo-labeled apoptotic germ cells by TM4 cells transfected with scramble (*Scr*) or *miR-471-5p* mimic (miR-471-5p) in the absence or presence of bafilomycin A1 (BFMY) (20 nM). **g** Immunofluorescence analysis showing phagocytosis of pHrodo-labeled apoptotic germ cells by TM4 cells transfected with scramble (*Scr*), *miR-471-5p* mimic, Dock180 expression vector, or cotransfected with *miR-471-5p* and Dock180 expression vector or *miR-471-5p* and Dock180–DHR2 mutant construct (mut Dock180). For both (**f**) and (**g**), Sertoli cells were stained with FITC-labeled actin (*green*) to show cell morphology. **h** Histogram showing percentage of Sertoli cells engulfing apoptotic germ cells as in groups described in **g**. The data are presented as mean ± SEM of three independent experiments (200 Sertoli cells counted per group per experiment). ***p = 0.001; ****p < 0.0001, one-way ANOVA followed by Tukey’s multiple comparisons test. *Scale bar* indicates 25 μm (**a**, **b**, **f**, **g**)

Next, we directly addressed whether *miR-471-5p* affected Sertoli cells’ ability to engulf and clear apoptotic germ cells. To address this, we first performed in vitro phagocytosis assays, where engulfment of apoptotic germ cells by Sertoli cells was observed as phagocytic cup (Supplementary Fig. 4a). To ensure that only internalized germ cells were counted, we labeled the apoptotic germ cells with a fluorescent dye, pHrodo, which glows only when apoptotic cells are internalized into the acidic environment of the phagolysosome^18^. Flow cytometry analysis and fluorescence microscopy showed that *miR-471-5p* mimic-transfected 15P1 Sertoli cells and primary Sertoli cells had significantly compromised ability to engulf pHrodo-labeled apoptotic germ cells as determined by reduced percentage of Sertoli cells taking up the apoptotic germ cells as well as reduced number of apoptotic germ cells engulfed per Sertoli cell (Fig. 3a–c and Supplementary Figs 4b, c, 5a). Consistent with that finding, primary Sertoli cells from miR-471-5pTg mice showed significantly impaired ability to internalize apoptotic germ cells (Fig. 3d, e). To further substantiate those findings, we performed phagocytosis assay in *miR-471-5p*-transfected cells in the presence and absence of bafilomycin A1, a lysosomotropic agent that halts the clearing process by inhibiting lysosomal degradation/autophagosome–lysosome fusion^19^. As expected, bafilomycin A1 treatment did not affect the engulfment of apoptotic germ cells by Sertoli cells, whereas *miR-471-5p* in the presence or absence of bafilomycin A1 inhibited uptake of apoptotic germ cells by Sertoli cells (Fig. 3f and Supplementary Fig. 5b). To ensure that impaired Sertoli cell engulfment of apoptotic germ cells is not due to compromised Sertoli cell development, we performed electron microscopy and observed no change in Sertoli cell morphology in miR-471-5pTg mice (Supplementary Fig. 6a). Next, we determined the level of acyl-CoA dehydrogenase, long chain (ACADL), which catalyzes β-oxidation of lipids in Sertoli cells and is dramatically increased when Sertoli cells phagocytose apoptotic germ cells^20^. We observed significantly decreased levels of ACADL in Sertoli cells of miR-471-5pTg in comparison with wild-type Sertoli cells (Supplementary Fig. 6b).

To address whether *miR-471-5p* regulated engulfment of apoptotic germ cells via Dock180, we performed rescue experiments. Primary Sertoli cells were transfected with Dock180 siRNA or co-transfected with *miR-471-5p* and Dock180 expression construct and subjected to phagocytosis assay using pHrodo-labeled apoptotic germ cells. The number of Sertoli cells engulfing apoptotic germ cells was significantly decreased in Dock180 siRNA transfected cells, while number of apoptotic germ cell containing Sertoli cells was significantly increased in *miR-471-5p* and Dock180 expression plasmid co-transfected primary Sertoli cells in relation to *miR-471-5p*-transfected primary Sertoli cells (Fig. 3g, h and Supplementary Fig. 6c). To address the specificity and to further substantiate the importance of Dock180 in *miR-471-5p*-mediated Sertoli cell phagocytosis, we performed phagocytosis assay using a mutant construct of Dock180–DHR2 domain, which is reported to be vital for Rac1 GTPase loading and activity during engulfment^4^. As expected, the Dock180–DHR2 domain mutant construct could not rescue *miR-471-5p*-mediated inhibition of germ cell engulfment (Fig. 3g, h). Because Rac1 GTPase is crucial for Dock180-mediated engulfment of apoptotic cells, we asked whether *miR-471-5p* also affects Rac1 activity. Indeed, we observed reduced levels of active Rac1 in *miR-471-5p* mimic-transfected Sertoli cells; however, the level of total Rac1 did not change (Supplementary Fig. 6d). Because Dock180, along with Elmo1, is crucial for germ cell engulfment by Sertoli cells^3^, we asked whether like Dock180, Elmo1 can rescue the effect of *miR-471-5p* overexpression on apoptotic germ cell engulfment. We observed no significant increase in germ cell phagocytosis in Elmo1 expression vector and *miR-471-5p* mimic co-transfected Sertoli cells in comparison with *miR-471-5p* mimic transfected cells (Supplementary Fig. 7). That result is not surprising given that Elmo1 requires Dock180, which is significantly reduced in miR-471-5pTg mice, to induce Rac1 GTPase and subsequently germ cell internalization^3^. Those results indicate that *miR-471-5p* regulates engulfment of apoptotic germ cells via Dock180–Rac1 GTPase signaling cascade.

### Sertoli cells use LAP for clearing apoptotic germ cells

**Fig. 4:**
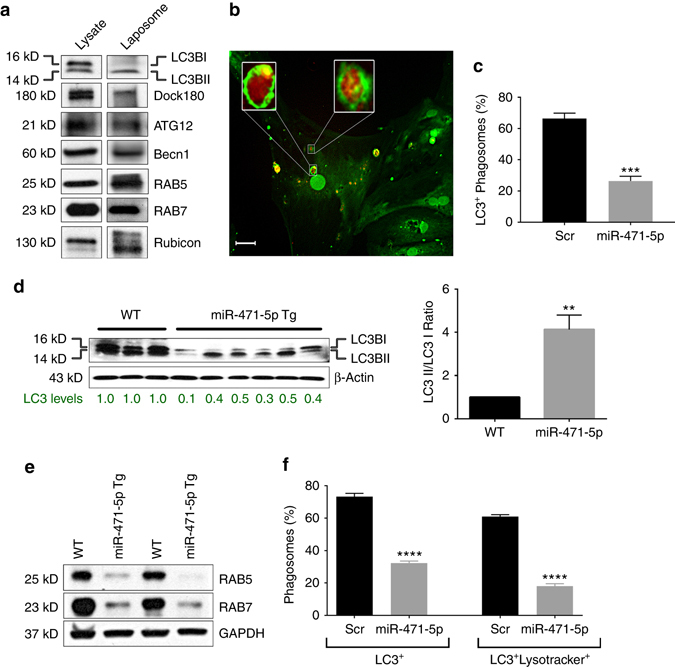
*MiR-471-5p* regulates LAP of apoptotic germ cells by Sertoli cells. **a** Western blot analysis on LC3 containing phagosomes (Laposome) isolated from the TM4 Sertoli cells fed with apoptotic germ cells using indicated antibodies. Gel photograph is representative of four independent experiments. **b** Immunofluorescence analysis showing LC3B (*green*) coating of phagocytozed apoptotic germ cell by the Sertoli cells. In vitro phagocytosis assay was performed using PI labeled apoptotic germ cell followed by immunofluorescence using LC3B antibody. *Inset* shows magnified view of the engulfed apoptotic germ cells coated by LC3B (*green*). **c** Bar graph showing percentage of LC3^+^ phagosomes containing PI-labeled apoptotic germ cells in scramble or *miR-471-5p* mimic transfected Sertoli cells as obtained by immunofluorescence analysis. The data represent mean ± SEM of three independent experiments (*n* > 100 per group), ***p = 0.0001, two-tailed unpaired student *t*-test. **d** *Left panel*, western blot analysis showing LC3 levels in normal control (WT) and miR-471-5pTg mice using antibody against LC3. Gel photograph is representative of three independent experiments (WT: *n* = 3 mice/experiment; miR-471-5pTg: 5 mice/experiment). Number below the gel represents band intensity quantified from all three experiments using Image J software. *Right panel*, histogram showing LC3II/I ratio derived from band intensities of western blot gels described in the *left panel* (WT: *n* = 3 mice per experiment; miR-471-5pTg: 5 mice per experiment), **p < 0.01; two-tailed unpaired student *t*-test. **e** Western blot analysis of miR-471-5pTg mice testis using antibodies against RAB5 and RAB7. Gel photograph is representative of three independent experiments (*n* = 3 for control and miR-471-5p Tg/experiment). **f** Histogram showing LC3^+^ and lysotracker^+^ LC3^+^ phagosomes from primary Sertoli cells transfected with either scramble or *miR-471-5p* mimic. Sertoli cells were preloaded with lysotracker blue and fed with pHrodo-labeled apoptotic germ cells followed by staining with antibody against LC3 before being subjected to fluorescence microscopy. Only those Sertoli cells that show engulfed apoptotic germ cells in scramble and *miR-471-5p* mimic transfected groups were counted (25 cells/experiment; *n* = 3 experiments). ****p < 0.0001, two-tailed unpaired student *t*-test. *Scale bar* indicates 10 μm (**b**)

After apoptotic germ cells are engulfed, the lysosome-dependent clearing process begins in the Sertoli cells^21, 22^. Phagosomes fuse with lysosomes, and the lysosomal hydrolases later degrade intraphagosomal components^5^. As *miR-471-5p* targets several autophagy component proteins that are known to play a role in recruiting LC3 to the phagosomal membrane during LAP^1, 6^, we set out to determine whether *miR-471-5p* could also regulate the clearing of apoptotic germ cells by LAP after they are engulfed. Moreover, miR-471-5p Tg mice serve as a good model to study both engulfment and clearance of germ cells as these mice don’t have complete blockage of germ cell engulfment by the Sertoli cells. To study LAP in Sertoli cells, we first determined whether proteins known to be associated with LAP in macrophages were also recruited to the LC3-containing phagosomes in the Sertoli cells. Indeed, phagosomes isolated from Sertoli cells showed recruitment of several proteins including LC3II, Beclin1, Atg12, rubicon, Rab5 and Rab7 (Fig. 4a). Interestingly, Dock180 was also observed to be recruited to LC3-containing phagosomes in Sertoli cells (Fig. 4a). Consistent with these findings, LC3 was observed to coat phagocytozed apoptotic germ cells in the primary Sertoli cells (Fig. 4b). Next, we assessed percentage of LC3-associated phagosomes in scramble and *miR-471-5p* mimic transfected Sertoli cells containing apoptotic germ cells. Immunofluorescence analysis of Sertoli cells fed with propidium iodide-labeled germ cells using antibody against LC3 showed that the percentage of LC3II^+^ phagosomes containing apoptotic germ cells was significantly reduced in *miR-471-5p* mimic transfected Sertoli cells when compared to scramble transfected Sertoli cells (Fig. 4c). To ensure that only membrane bound LC3 (LC3II) associated with phagosomes were counted, we treated Sertoli cells with digitonin, which depletes LC3I^6^. To further support the effect of *miR-471-5p* on Sertoli cell LAP, we determined the effect of *miR-471-5p* on LC3 levels in the Sertoli cells. MiR-471-5p Tg mice showed significantly reduced levels of LC3 protein and altered LC3II/I ratio (Fig. 4d).

LC3-II is reported to be critical for LC3-containing phagosome maturation^6^. To address whether *miR-471-5p* can also regulate LC3-containing phagosome maturation, we examined levels of GTPase Rab5, which is recruited to the LC3-containing phagosome by Becn1 and acts as a critical regulator of LC3-containing phagosomal maturation and clearance of apoptotic cells during LAP^6^. Interestingly, Sertoli cells from miR-471-5p Tg mice showed significantly lower levels of Rab5 when compared to normal controls (Fig. 4e). In addition, level of Rab7, which acts downstream of Rab5 and is known to localize to phagosomes^14, 23^, was significantly lower in miR-471-5p Tg mice compared to normal control (Fig. 4e). To further substantiate these results, Sertoli cells were transfected with scramble or *miR-471-5p* mimic and preloaded with lysotracker blue, which is used to monitor acidification and function of lysosome, and subsequently fed with pHrodo-labeled apoptotic germ cells. *miR-471-5p mimic*-transfected Sertoli cells showed impaired LC3 and lysotracker recruitment around phagocytozed germ cells (Fig. 4f and Supplementary Fig. 8). Taken together, those findings indicated that *miR-471-5p* plays an important role in LAP-mediated clearance/degradation of apoptotic germ cells.

### Dock180 is directly involved in Sertoli cell LAP

**Fig. 5:**
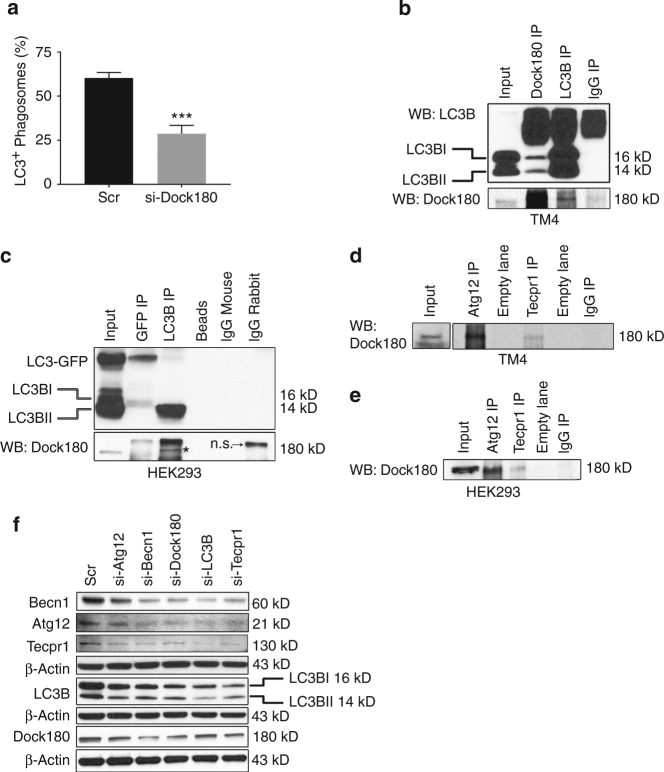
Dock180 interacts with LC3B and other autophagy-associated proteins to regulate LAP in Sertoli cells. **a** The percentage of LC3^+^ phagosomes containing apoptotic germ cells in scramble-siRNA or Dock180-siRNA transfected Sertoli cells as obtained from immunofluorescence analysis. The data represent mean ± SEM of three independent experiments (*n* = 100 per group), ***p = 0.0006, two-tailed unpaired student *t*-test. **b**, **c** Dock180 interacts with LC3 in mammalian cells. Immunoprecipitation on TM4 cells (**b**) or HEK293 cells (**c**) using antibodies against Dock180 (Dock180 IP), LC3B (LC3B IP), GFP (GFP IP), rabbit IgG (IgG IP) or mouse IgG (IgG mouse) (**c**) and probed (WB) with LC3B or Dock180 antibodies. For **c**, cells were transfected with GFP-LC3B-RFP expression construct before being subjected to immunoprecipitation. Immunoprecipitation with beads alone (Beads) served as a negative control. *Asterisk* in **c** indicates Dock180 band. n.s represents ‘non-specific’ band. **d**, **e** Immunoprecipitation on TM4 (**d**) and HEK293 (**e**) using antibodies against Atg12 (Atg12 IP) or Tecpr1 (Tecpr1 IP) or rabbit IgG (IgG IP) and probed with Dock180 antibody. **f** Western blot analysis on TM4 Sertoli cells transfected with scramble (Scr) siRNA or siRNAs (si) against autophagy associated proteins Becn1 (si-Becn1), Atg12 (si-Atg12), Tecpr1 (si-Tecpr1), and LC3B (si-LC3B) using antibody against indicated autophagy proteins. β-Actin served as a loading control. Gel photograph is representative of four independent experiments. Bar graphs showing quantification of band intensities are shown in Supplementary Fig. 10

Having shown that Dock180 is highly expressed in Sertoli cells containing LC3^+^ phagosomes, we wondered whether Dock180 is directly involved in the Sertoli cell LAP. Surprisingly, like *miR-471-5p*, Dock180 silencing decreased the percentage of LC3II^+^ phagosomes compared to scramble-transfected Sertoli cells (Fig. 5a). That finding is significant because it suggested that in addition to its well-established role in engulfment, Dock180 plays an equally important role in LAP-mediated clearance of apoptotic germ cells. To understand how Dock180 regulates LAP, we tested whether Dock180 functionally/physically interact with LC3 and other autophagy-associated proteins. To our surprise, immunoprecipitation experiments showed that Dock180 interacts with LC3 in Sertoli cells (Fig. 5b). Dock180 interaction with LC3 was not restricted only to Sertoli cells; it also showed interaction in other mammalian cells, indicating that Dock180 plays a broader role in LC3-associated clearance of cellular debris in mammalian cells (Fig. 5c and Supplementary Fig. 9). Furthermore, immunoprecipitation experiments revealed that Dock180 interacts with Atg12 and Tecpr1 in Sertoli cells as well as in other mammalian cells (Fig. 5d, e). These results suggested that Dock180 and some autophagy-associated proteins constitute a functional phagocytic complex that plays a crucial role in clearance of germ cells by LAP. Supporting these results, we found that silencing either Dock180 or LC3B/Atg12/Tecpr1/Becn1 decreased the levels of other proteins (Fig. 5f and Supplementary Fig. 10a–e).

### Androgen regulates engulfment of germ cells via miR-471-5p

**Fig. 6:**
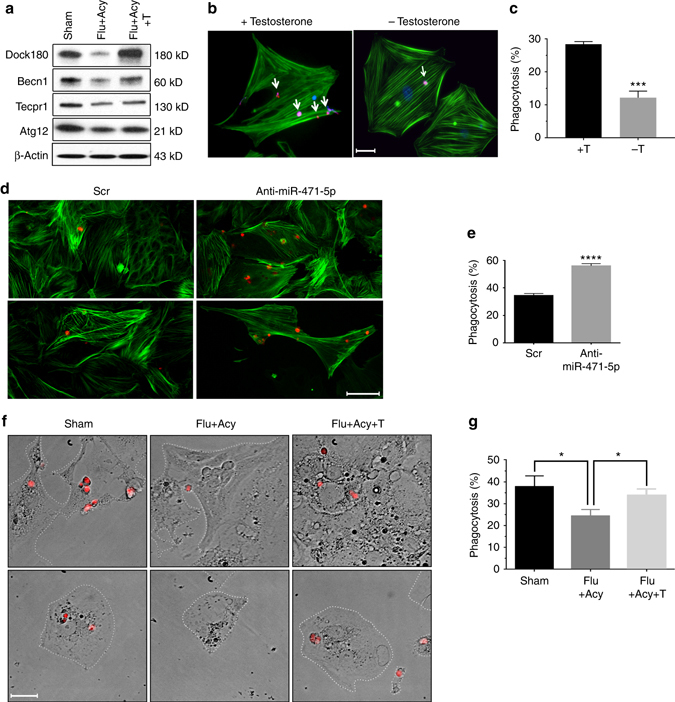
Androgen regulates Sertoli cell phagocytosis of apoptotic germ cells via *miR-471-5p*-Dock180/autophagy protein signaling pathway. **a** Western blot analysis on enriched Sertoli cells isolated from Sham, flutamide–acyline (Flu+Acy), and flutamide-acyline-testosterone (Flu+Acy+T)-treated mice testes using antibodies against Dock180, Atg12, Tecpr1, and Becn1. β-Actin was used as a loading control. Gel photograph is representative of two independent experiments; Sertoli cells were pooled from 7 mice per experiment. Bar graphs showing quantification of band intensities are shown in Supplementary Fig. 11a. **b** Immunofluorescence analysis showing number of pHrodo-labeled apoptotic germ cells engulfed by Sertoli cells in the presence (+) and absence (−) of testosterone. **c** Histogram showing number of Sertoli cells engulfing apoptotic germ cells in the absence (−T) and presence (+T) of testosterone as derived from the flow cytometry analysis. The data are presented as mean ± SEM of four independent experiments. ****p < 0.0001; two-tailed unpaired student *t*-test. **d** Immunofluorescence analysis showing engulfment of pHrodo-labeled apoptotic germ cells by primary Sertoli cells isolated from flutamide/acyline (Flut+Acy)-treated mice and cultured in charcoal-stripped medium and transfected with scramble (*Scr*) and *miR-471-5p* antagomiR (Anti-miR-471-5p). **e** Histogram showing number of primary Sertoli cells engulfing germ cells in experimental groups described in **d**. The data are presented as mean ± SEM of three independent experiments. ****p < 0.0001, two-tailed unpaired student *t*-test. **f** Immunofluorescence analysis showing in vitro phagocytosis by purified primary Sertoli cells from Sham, flutamide-acyline-treated (Flu+Acy), and flutamide-acyline-treated and supplemented with testosterone (Flut+Acy+T) mice. Purified Sertoli cells were cultured for 48 h in complete media, charcoal-stripped media, or charcoal-stripped media supplemented with testosterone before being subjected to phagocytosis assay. Sertoli cells are marked with *white dotted line* to clearly indicate outer border. **g** Histogram showing percentage of Sertoli cells from Sham, Flu+Acy, and Flu+Acy+T-treated mice engulfing apoptotic germ cells as derived from flow cytometry analysis. The data are presented as mean ± SEM of three independent experiments. *p < 0.01, one-way ANOVA followed by Tukey’s multiple comparisons test. *Scale bar* indicates 25 μm (**b**, **f**); 20 μm (**d**)

**Fig. 7:**
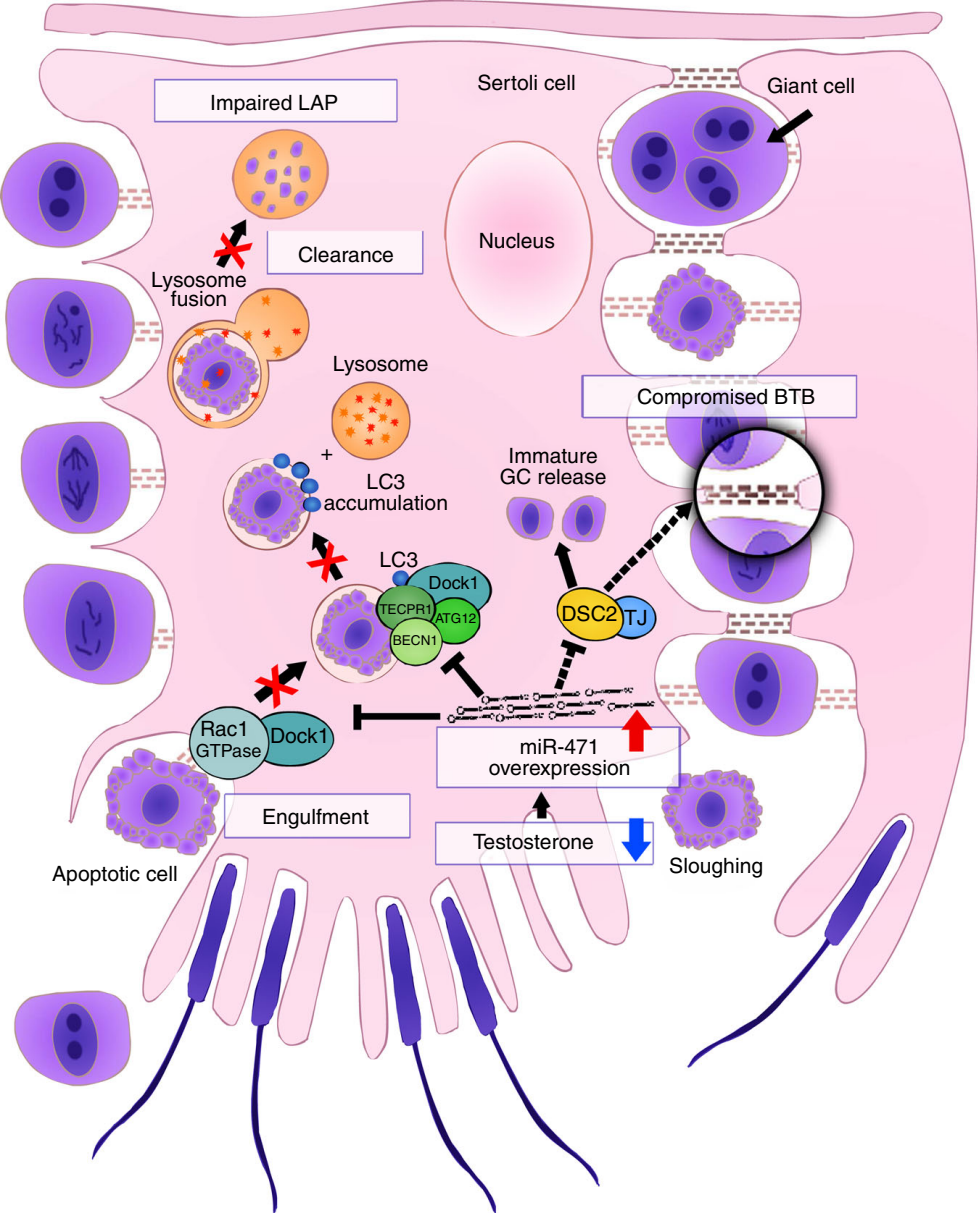
Role of androgen-responsive *miR-471-5p* in Sertoli cell. Model showing *miR-471-5p* regulation of Sertoli cell functions. We posit that lower than normal levels of androgen will increase *miR-471-5p* expression in Sertoli cells, leading to inhibition of target genes including *Dock1 (Dock180)*, *Atg12*, *Tecpr1*, and *Becn1*. The decreased levels of *Dock180* and other autophagy-associated genes in turn impairs LAP by inhibiting engulfment and/or phagolysosome maturation and consequently clearance of apoptotic germ cells by the Sertoli cells. In addition to LAP-associated proteins, miR-471-5p also regulates expression of BTB associated proteins, including Dsc2. Since Dsc2 is an integral component of desmosome junction, which is the primary attachment site for developing germ cell, it is possible that *miR-471-5p* promotes immature release of germ cells and consequently their apoptosis via Dsc2. Taken together, our results suggest that maintaining an optimal level of key genes, which are important for engulfment and clearance of apoptotic germ cells as well as BTB integrity in Sertoli cells, is critical for proper germ development and differentiation and male fertility. GC- Germ cell

Because *miR-471-5p* expression is androgen dependent^9^, we tested whether androgen also regulates *miR-471-5p* target genes and engulfment of apoptotic germ cells by the Sertoli cells. To address that question, we first determined levels of Dock180, Atg12, Becn1, and Tecpr1 in a flutamide-acyline-treated androgen suppression mouse model^9^. Levels of those proteins were significantly reduced in the absence of androgen, whereas testosterone supplementation restored levels of Dock180 and Becn1 close to those in normal controls in purified Sertoli cells (Fig. 6a and Supplementary Fig. 11a). Though levels of Tecpr1 and Atg12 showed significant decrease in anti-androgen-treated mice, the testosterone supplementation didn’t completely restore their levels to the control levels. This could be due several reasons: first, the effect of anti-androgen could be strong due to engagement of repressors on the gene promoter and testosterone supplementation is not be able to completely disengage the repressor complex; and second, the duration used for testosterone supplementation is not be long enough to see the rescue. Nevertheless, decreased levels of these genes in anti-androgen-treated groups clearly reflect that these genes are under androgen regulation. Next, we inquired whether androgen directly regulates expression of the corresponding genes. Bioinformatics analysis of the 5′-UTR of those genes revealed no putative androgen response elements indicating that androgen regulates expression of these genes via *miR-471-5p*. However, we cannot exclude the possibility that androgen is directly involved in regulating these genes as androgen receptor has non-genomic action^24^. Next, to test whether germ cell engulfment is an androgen-dependent event, we performed phagocytosis assay in the presence and absence of testosterone. Percentage of Sertoli cells engulfing apoptotic germ cells as well as number of engulfed germ cells/Sertoli cell significantly increased when primary Sertoli cells from normal control mice was cultured in the presence of testosterone compared to Sertoli cells cultured in charcoal stripped medium without testosterone, as revealed by flow cytometry and immunofluorescence microscopic analyses (Fig. 6b, c and Supplementary Fig. 11b). To further substantiate those results, we performed rescue experiments using *miR-471-5p* antagomiR. *miR-471-5p* expression is induced in the absence of androgen;^9^ therefore, we reasoned that primary Sertoli cells isolated from flutamide-acyline-treated mice and subsequently, cultured in charcoal-stripped medium would have increased levels of *miR-471-5p* and consequently decreased Sertoli cell phagocytosis. As a result, *miR-471-5p* antagomiR should rescue that effect. Indeed, primary Sertoli cells isolated from flutamide-acyline-treated mice and subsequently cultured in charcoal-stripped medium and later transfected with *miR-471-5p* antagomiR internalized significantly more apoptotic germ cells than did scramble-transfected Sertoli cells (Supplementary Fig. 11c). In addition, number of Sertoli cells engulfing apoptotic germ cells was significantly more in *miR-471-5p* antagomiR group when compared to scramble-transfected group (Fig. 6d, e). Consistent with that finding, the level of *miR-471-5p* was significantly higher in primary Sertoli cells from androgen suppressed mice and cultured in charcoal-stripped medium than in primary Sertoli cells from sham-treated mice and cultured in complete media (Supplementary Fig. 11d). To further support that assertion, we compared the ability of Sertoli cells to phagocytose apoptotic germ cells in control mice, mice treated with flutamide–acyline, and mice both treated with flutamide–acyline and supplemented with testosterone. We observed decreased phagocytosis in flutamide–acyline-treated Sertoli cells, whereas testosterone supplementation restored the level of phagocytosis as revealed by flow cytometry analysis (Fig. 6f, g). To further substantiate the notion that androgen indeed plays a role in phagocytosis, we compared levels of ACADL in control and flutamide-acyline-treated mice. We observed a significant decrease in ACADL level in flutamide-acyline-treated mice in comparison with levels in control groups (Supplementary Fig. 11e). Taken together, these findings suggest that *miR-471-5p* by regulating the expression of its target proteins including Dock180 and autophagy-associated proteins in Sertoli cells regulates LAP-mediated clearance of apoptotic germ cells, meiotic progression of developing germ cells, blood-testis barrier integrity and energy metabolism, which are vital for proper germ cell development and differentiation (Fig. 7).

## Discussion

Increasing evidence suggests that phagocytosis is not simply a waste clearance process but rather serves important cellular functions. For example, clearance of apoptotic cells by macrophages helps in their immune surveillance function^14^. Similarly, evidence continues to indicate that phagocytic clearance of apoptotic germ cells and residual bodies by Sertoli cells facilitates proper germ cell development and differentiation and ultimately efficient sperm production^20^. Although why so many germ cells die during spermatogenesis is not clear, phagocytic events clearly are rapid and efficient because not many apoptotic germ cells are seen at any given spermatogenic stage in the normal testis. Although several molecules that regulate the phagocytic process have been identified, our understanding of the mechanisms that regulate discrete steps of this orderly event is limited. Our studies suggest that interaction between androgen-responsive *miR-471-5p* and phagocytosis/autophagy-associated proteins regulates germ cell engulfment and as well as clearance using LAP. In support of *miR-471-5p*-mediated germ cell engulfment, we show that levels of Dock180 and consequently activity of Rac1-GTPase are significantly reduced in *miR-471-5p* mimic transfected Sertoli cells and miR-471-5p Tg mice. Furthermore, we show that Dock180 expression rescued phagocytosis of apoptotic germ cells by Sertoli cells ectopically expressing *miR-471-5p*. Since Elmo1 was recently shown to interact with Dock180 to induce Rac1-GTPAse^3^, leading to efficient engulfment of germ cells by Sertoli cells, our study introduces a new regulatory player: *miR-471-5p* in the Dock180–Elmo1–Rac1 signaling cascade during engulfment of apoptotic germ cells by the Sertoli cell. In support of the importance of *miR-471-5p* in the clearance of apoptotic germ cells, we show that *miR-471-5p* overexpression inhibits LC3-II recruitment to LC3-containing phagosomes. In addition, our study revealed that several autophagy-associated proteins are targets of *miR-471-5p* and are highly expressed in Sertoli cells. Furthermore, we demonstrate that Dock180 interacts with LC3 and other autophagy-associated proteins to regulate LAP-mediated clearance of apoptotic cargo in Sertoli cells.

Recruitment of LC3II to the phagosome containing apoptotic germ cells by autophagy-associated proteins is one way that the autophagy pathway can contribute to Sertoli cell LAP. Reduced levels of Dock180, LC3, Becn1 and Atg12 in our miR-471-5pTg mice support this notion. Furthermore, Dock180 interaction with LC3, whose lipidation and recruitment to the LC3-containing phagosome is regulated by Atg12/Tecpr1/Becn1, suggests that a functional complex of Dock180/LC3B and autophagy member proteins is vital for efficient clearance of apoptotic germ cells by Sertoli cells. Consistent with that assertion, knockout of *Atg5* reduced the levels of LC3II surrounding the phagosome^25^. In addition to LC3II recruitment, autophagy component proteins can be instrumental in the maturation of the LC3-containing phagosome during Sertoli cell LAP. In support of that assertion, autophagy component proteins Becn1 and the Atg5/12/16 complex are required for LAP of dead cells by macrophages^1^. Furthermore, Becn1 is reported to be instrumental in recruiting Rab5, which is a critical mediator of phagosomal maturation and clearance of apoptotic cells during LAP^6^. Moreover, our results show that *miR-471-5p* targets Rab5/Rab7 and regulates maturation of LC3-contianing phagosomes. LAP in macrophages involves identification of phosphatidylserine (Ptdser), which is exposed extracellularly on the dying cells and serves as an “eat me” signal, by the TIM4 receptor^3^. However, TIM4 is reportedly not expressed in Sertoli cells, suggesting that Sertoli cells recruit other receptors to engage Ptdser on apoptotic germ cells for LAP^5^. An example of those receptors is BAI1 (brain angiogenesis inhibitor 1), which is a transmembrane protein highly expressed in Sertoli cells that plays an important role in Sertoli cell phagocytosis^3^. Future studies will unveil how BAI1 or other receptors expressed in Sertoli cells connect to the Becn1 or Atg5/Atg12-associated signaling involved in LAP in Sertoli cells.

The conditional knockout of the androgen receptor in Sertoli cells and other approaches have clearly shown that androgen is necessary to complete germ cell meiosis and spermatid differentiation^26, 27^. Our study adds a new role for androgen in the testicular physiology as it shows phagocytosis of apoptotic germ cells by Sertoli cells to be an androgen-dependent event. Reduced levels of Dock180 and the autophagy proteins Atg12, Tecpr1, and Becn1 in mice treated with flutamide-acyline indicate that androgen regulates both the engulfment and the clearance of apoptotic germ cells in Sertoli cells. Further supporting the notion that androgen is directly associated with phagocytosis of apoptotic gem cells is our finding that androgen deprivation reduced levels of lipid B-oxidation enzyme ACADL. Because phagocytosis of dying germ cells acts as a source of lipid availability to Sertoli cells for germ cell development, and with lipid oxidation being the predominant pathway for ATP production in Sertoli cells^20, 28^, androgen probably plays an important role in Sertoli cell energy metabolism by regulating phagocytic process. The association between phagocytosis of apoptotic germ cells by Sertoli cells and germ cell development is significant given that ATP production in Sertoli cells is highest among the cells including macrophages that have phagocytic ability^20^. Supporting that notion, androgen receptor-knockout mice had reduced levels of ACADL in muscle^29^. In contrast to our findings, an earlier report^30^ suggested that androgen may not be directly involved in phagocytosis of germ cells. That conclusion was based solely on microscopic observation of apoptotic germ cells in Sertoli cells in androgen-suppressed rat testis. That assertion does not accurately reflect how androgen affects the rate of phagocytosis as Sertoli cells in androgen-suppressed mice will have access to substantially more apoptotic germ cells to phagocytose. To accurately compare the rate of phagocytosis, an equal number of apoptotic germ cells must be fed to Sertoli cells isolated from both control and androgen-suppressed mice, as used in our studies.

In summary, we show that maintaining an optimal level of key factors such as Dock180 and autophagy/phagocytosis-associated proteins is vital for engulfment and clearance of cellular debris by mammalian cells in general and Sertoli cells in particular. Our findings offer compelling evidence that LAP is critical for efficient clearance of apoptotic germ cells, which is necessary to meet high-energy (ATP) needs of Sertoli cells for maintaining germ cell development and differentiation. In addition, since phagocytic clearance of apoptotic germ cells by Sertoli cells is essential for maintaining testicular homeostasis, our study showing mechanisms that regulate discrete steps of the phagocytic process in Sertoli cells will be vital not only to ensure spermatogenic success but also to avoid autoimmune responses such as orchitis, an etiological factor of male infertility^31^.

## Methods

### Animals and reproductive phenotype analyses

All animal experiments were performed in accordance with the National Institutes of Health Guide for the Care and Use of Laboratory Animals. Approval of animal use for this study was granted by the Institutional Animal Care and Use Committee of the University of Texas Health Science Center at San Antonio (Animal Welfare Assurance #A3345-01; Protocol #07057-34-02-A). For sperm count, 2- to 3-month-old male mice were euthanized by CO_2_, caudal epididymides were harvested, small cuts were made in the epididymides, and sperm were allowed to disperse into the medium for 15 min at 37 °C. Sperm was diluted 1:10 before counting on a hemocytometer. Tissue preparation, 8-week timed mating, hematoxylin and eosin staining, and TUNEL assay were conducted using standard techniques^17^.

### Generation of miR-471-5p transgenic mice

To generate miRNA transgenic mice, we used PEM-236 plasmid^10^, which includes 0.6 kb Sertoli cell-specific *Rhox5* proximal promoter and bovine growth hormone 3′-UTR and polyadenylation signal (BGH-pA). Primary miRNA sequence that includes ~ 400-bp sequence flanking the mature mmu-miR-471-5p was cloned in PEM-236 vector, further confirmed by the DNA Sequencing core facility at UTHSCSA. MiR-471-5pTg mice (B6D2F1 strain) were generated at the University of Texas M. D. Anderson Cancer Center Genetically Engineered Mouse Facility, Houston, by liberating the transgene from the plasmid backbone by digestion with Sall and NdeI and then injecting this DNA into mouse pronuclei. Ten founder transgenic lines were generated, as detected by PCR genotyping using tail genomic DNA as a template.

### Electron microscopy

Animals were perfused and testis were harvested and fixed in 1% glutaraldehyde followed by electron microscopic sectioning^17^. Electron microscopic sectioning was performed in the EM core facility at UTHSCSA.

### Sperm abnormalities

To analyze the sperm abnormalities in the wild-type and miR-471 Tg mice, caudal spermatozoa were dispersed and fixed in 4% paraformaldehyde and smeared in the microscopic grade slides and observed under microscopy^17^.

### Rac1 activation assay

GTP loading of Rac1 was measured using the Rac1 Activation Assay Kit (Millipore, Massachusetts, USA) and Thermo scientific (cat. #16118) according to the manufacturer’s instructions. In brief, TM4 Sertoli cells were transfected with miR-471 mimic for 48–72 h and cells were lysed in ice-cold magnesium lysis buffer. Cell extracts were then incubated with PAK1–PBD agarose beads, pelleted, and washed. The beads were resuspended in 2× Laemmli sample buffer and separated by sodium dodecyl sulfate 4–15% polyacrylamide gel electrophoresis. GTP-bound Rac1 was detected using an anti-Rac1 monoclonal antibody (Millipore, Massachusetts, USA). An aliquot of lysate retained before pull-down assay was subjected to immunoblotting to determine total Rac1. Representative immunoblots were scanned and subjected to densitometry to compare activated with total GTPase activity.

### siRNA transfection

Atg12, Becn1, Dock180, LC3B, Tecpr1, and scramble siRNA were purchased from Sigma & Santa Cruz Biotechnology. TM4, 15P1, and primary Sertoli cells were transfected using Lipofectamine RNAi max (Life Technology). At 48 and 72 h after transfection, cells were harvested and analyzed for RNA and protein.

### Sertoli cell culture and Sertoli cell lines

For Sertoli cell culture, adult mice testes were removed and decapsulated in HBSS medium^24, 9^. Testicular seminiferous tubules were placed in the 50 ml conical flask containing 50 ml HBSS containing 0.5 mg/mL, in a collagenase for 10–15 min at 34 °C in the constant shaker. After cells were allowed to settle down and the supernatant containing Leydig/interstitial cells were removed. The supernatant, which contained interstitial cells, was decanted. Resulting seminiferous tubules were washed 3 times with HBSS medium. The seminiferous tubules were incubated with 0.5 mg/ml trypsin solution for 10 min, at 37 °C, without shaking. Then seminiferous tubules were incubated with trypsin inhibitor (0.3 mg/ml) for 5 min. To separate Sertoli cells from germ cells, seminiferous tubules were treated with collagenase, hyaluronidase and DNase I and trypsin inhibitor for 45 min at 34 °C in the shaker. Then Sertoli cell prep was centrifuged at 500 rpm for 5 min for 3 times. To get pure Sertoli cells, the prep was incubated with hypotonic shock solution (1 ml HBSS:10 Deionized water) for 1 min and centrifuged at 500 rpm for 4 min. Pure Sertoli cells were filtered through 40 μm pore-size nylon mesh and Sertoli cells were cultured in 6 well plates at 34 °C in the complete DMEM medium containing 10% FBS. For androgen-related experiemnts, primary Sertoli cells were grown in charcoal-stripped serum containing medium. Sertoli cell lines 15P1 and TM4 were cultured in RPMI 1640 and DMEM, respectively, as per ATCC reccomendations. Sertoli cell lines were checked for mycoplasma contamination and were authenticated using antibodies aginast Sertoli cells-specific proteins Sox9 and Amh.

### Immunohistochemistry and western blot analysis

We carried out immunohistochemical analyses on 4% paraformaldehyde-fixed and paraffin-embedded testicular sections^9^. We performed western blot analysis on testis extracts from wild-type, miR-471 Tg, sham control (Sham), flutamide-acyline (Flu+Acy), and androgen-replacement (Flu+Acy+T) groups; or cell extracts from miR-471 mimic-transfected 15P1 and TM4 Sertoli cells lines and primary Sertoli cells^9^. We purchased antibodies to horseradish peroxidase-conjugated β-actin (1:50,000; Sigma-Aldrich, cat#A5316), β-tubulin (Sigma; T8328), DOCK180 (1:1000; Sigma-Aldrich; D9820), ACADL (1:1000; Sigma, #SAB2100020), TECPR1(1:1000; Abgent #AP7391b-ev), and Atg12 (1:1000; #A8731) from Sigma Inc. Antibodies for Sox9 (1:1000; Millipore, cat#AB5535), GAPDH(1:25,000; Sigma, Cat #G9295) and LC3B (1:3000; Sigma, L7543) were purchased from Millipore. Antibody to DOCK180 (1:1000; Thermo Scientific #MA5-15010) and Becn1 (1:1000; SC-11427) was purchased from Santa Cruz. Antibody to Ago2 was purchased from Wako Chemicals, Richmond, VA, USA (1:200; Cat #018-22021). RAB5 (1:1000; cat #2143), RAB7 (1:1000; cat#9367), Rubicon (1:1000; cat#8465), LC3A/B (1:2000; CST; cat #12741), cleaved caspase 3 (1:200; cat #9661) and Atg12 (1:1000; cat #418) antibodies were purchased form cell signaling technology. Antibody to Ago2 was purchased from.

### Plasmids

Luciferase 3′-UTR segments of the *DOCK180* gene were PCR amplified and subcloned into the pMIR-REPORT vector (Life Technologies) at the SacI and SpeI restriction sites. Sertoli cells or primary Sertoli cells were transfected with miR-471-5p mimic or scramble miRNA (Invitrogen, USA). After 24 h cells were again transfected with Dock180-overexpressing plasmid (gift from Prof. Kristiina Vuori, Sanford-Burnham Medical Research Institute, La Jolla, CA), and 48 h after transfection, cells were used for phagocytosis assay. MAP1LC3B and ELMO1 plasmids were purchased from Origene (MAP1LC3B; cat #MG200654; ELMO1 cat #MR206637).

### Blood–testis barrier assay

WT and miR-471 Tg mice were anesthetized and testes were exposed and injected with biotin tracer dye (EZ-Link NHS-LC-Biotin; Cat#21336, Thermo Scientific, USA). After 30 min of biotin tracer dye injection, testes were harvested, fixed in 4% formaldehyde and sectioned at 5μM thickness. Testis sections were deparafinized in xylene (10 min x 2 times) and rehydrated using alcohol gradient (5 min each in 100%, 95%, 90%, 70%, 50%, 30%) followed by PBS for 5 min. Testes sections were permeabilized with 0.01% Troton X and blocked with 10% normal goat serum followed by incubation with FITC-conjugated streptavidin (1:200; cat #1001, Invitrogen) for 1 h in dark^16, 32^.

### Phagocytosis assay

Sertoli cell lines TM4, 15P1 or primary Sertoli cells were transfected with scramble, *miR-471-5p* mimic, Anti-miR-471-5p, Dock180 expression vector, Dock180–DHR2 domain mutant construct, or Dock180-siRNA for 48 h followed by incubation with pHrodo Red dye (cat. #P36600; Life Technologies)-labeled germ cells^3, 18^. Before germ cells were labeled with pHrodo Red dye, germ cells were harvested from the control mice and induced to undergo apoptosis^3^, which was confirmed by annexin V staining. Apoptotic germ cells were then labeled with pHrodo dye for 1 h at room temperature in the dark, followed by washing and incubation with primary Sertoli cells, TM4, or 15P1 Sertoli cells. After 3 h of co-incubation, engulfed apoptotic germ cells were observed under microscope or were subjected to flow cytometry analysis. Phagocytosis assay for bafilomycin A1-treated cells was performed by transfecting TM4 cells with scramble or *miR-471-5p* mimic for 48 h followed by bafilomycin A1 treatment at 10–100 nM concentration (Sigma #B1793) and incubation with pHrodo dye-labeled germ cells as described above. Phagocytic flux assays for bafilomycin A1-treated groups were performed in TM4 cells transfected with scramble or *miR-471-5p* in the presence of bafilomycin A1.

### Phagosome isolation

TM4 Sertoli cells were incubated with mixture of apoptotic germ cells and protein A magnetic beads for 2 h. Cells were then homogenized in buffer containing 250 mM sucrose and 3 mM imidazole (pH 7.4) and a part of the homogenized sample was stored as input (lysate) and rest was used to isolate phagosome^5, 33^. Phagosomes were subjected to SDS-PAGE western blot analysis. To study the effect of *miR-471-5p* on LC3 positive phagosomes, TM4 cells were transfected with scramble or *miR-471-5p* mimic for 48 h followed by incubation with PI-labeled apoptotic germ cells for 2 h. Cells were then trypsinized, treated with digitonin for 15 min followed by staining with antibody against LC3B and then FITC-labeled secondary antibody to localize the endogenous LC3II. The percentage of LC3^+^ apoptotic germ cell containing phagosomes were calculated using immunofluorescence analysis^1^.

### Ago2 pull-down and RNA Seq

To identify miR-471-5p target genes, cells were transfected with either scramble or *miR-471-5p* mimic and after 48 h, cells were subjected to RNA immunoprecipitation using Ago2 antibody. RNA-protein complexes were digested with proteinase K and RNA was subjected to RNA deep sequencing (Illumina).

### Statistical analysis

All values and error bars in graphs are means ± standard error of the mean; respective *n* values are indicated in figure legends; *p* values were determined by Student’s *t*-tests and analysis of variance (ANOVA). For studies involving mice, sample size is estimated by using t-test or Wilcoxon test with mean difference from constant (one group) or between each other (two groups). For example, for Fig. 1d, reference to WT of 2 litters/2 months, and considering the number of litters from the transgenic line are possible 0, 1, and 2, we estimated, assuming a discrete uniform distribution, the standard deviation is ~ 0.82 (or effective size of 1.22). Using Wilcoxon test, we estimated at least 6 transgenic mice are required to reach statistical power of 80% with significance at 0.05. Similar assumption were made for number of pups (Fig. 1e) and sperm count (Fig. 1f). Sample size calculations were performed using PASS 14 (NCSS, LLC, UTAH). For animal experiments, researcher were blinded to the miR-471-5p transgenic lines and treatment groups. Animals were randomized for all experiments.

### Data availability

The data set for Ago2 pull down assay is submitted to NCBI (GSE99500). The authors declare that all other relevant data supporting the findings of this study are available within the article and its Supplementary Information files or from the corresponding author upon request. All other data that support the findings of this study are available from the corresponding author upon request.

## Electronic supplementary material

Supplementary Information
