# Supplementary material for: Cross-talk between miR-471-5p and autophagy component proteins regulates LC3-associated phagocytosis (LAP) of apoptotic germ cells
Source: Nat Commun. 2017 Sep 19;8:598. doi: 10.1038/s41467-017-00590-9 (PMC5605700; doi:10.1038/s41467-017-00590-9)
Supplement: Supplementary file 1 — Supplementary Information [file 41467_2017_590_MOESM1_ESM.pdf]

Description of Supplementary Files

File name: Supplementary Information
Description: Supplementary figures and supplementary tables.

Supplementary Figure 1

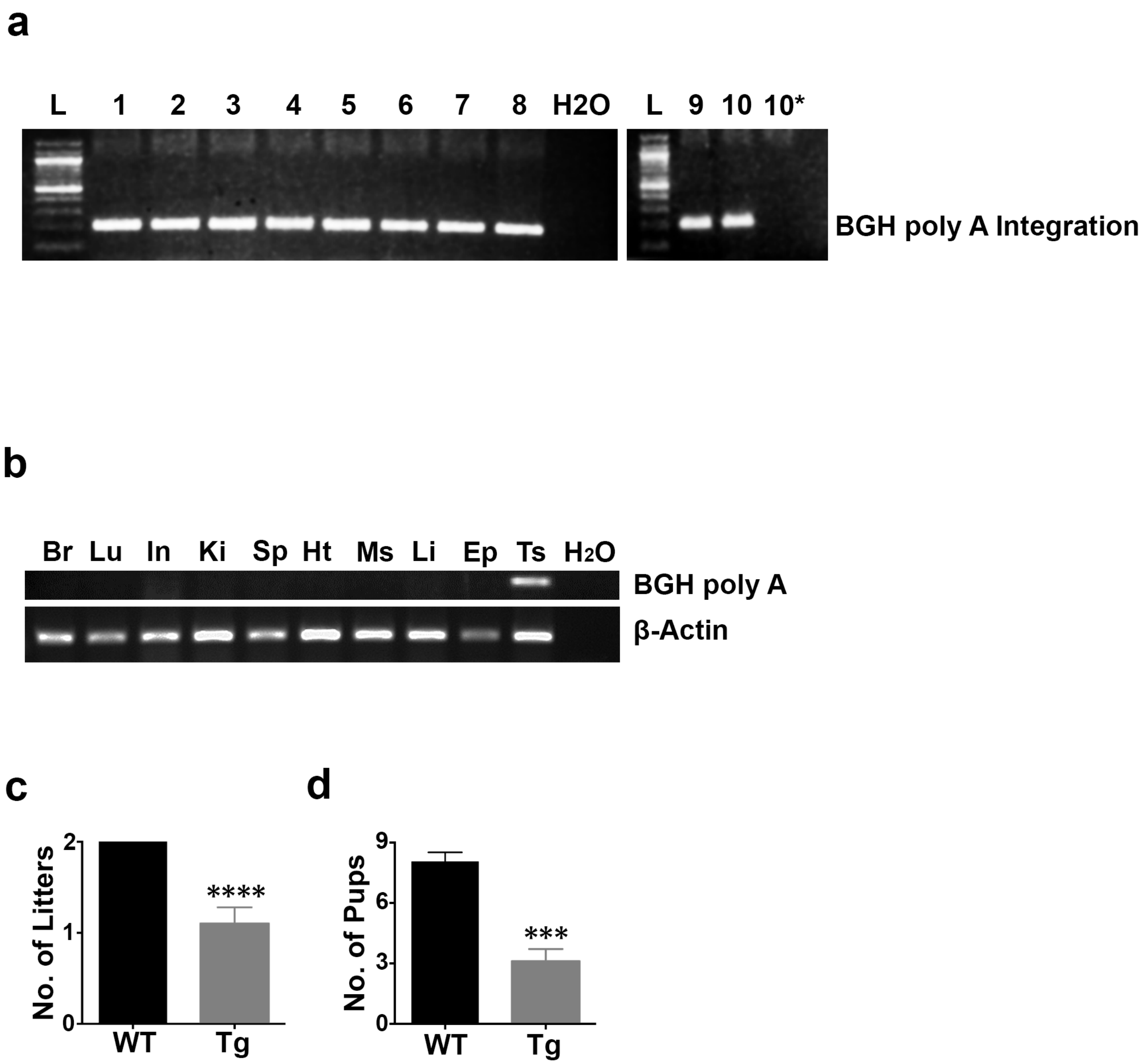

Supplementary Figure 1. Generation of miR-471-5p BGH polyA transgenic mice. (a) PCR analysis using genomic DNA (gDNA) and BGH polyA-specific primers showing transgene integration in 10 miR-471-5p transgenic lines. Lane 10* represents tail gDNA without BGH polyA primer. (b) RT-PCR analysis on total RNA from miR-471-5p Tg mice vital organs using BGH polyA-specific primers (top panel). Br=Brain; Lu=Lung; In=Intestine; Ki=Kidney; Sp=Spleen; Ht=Heart; Ms=Muscle; Li=Liver; Ep=Epididymis; Ts=Testis; H2O=Water. β-Actin was used as a loading control (bottom panel). (c, d) Total number of litters (c) and pups per litter (d) obtained from 2 months matings between control male and control female (WT) and between miR-471-5pTg male (from all 10 transgenic lines) with control female littermates (n = 10 for WT; and n = 10 for each of the 10 miR-471-5p transgenic lines). ****p< 0.0001, two tailed unpaired student t-test.

Supplementary Figure 2

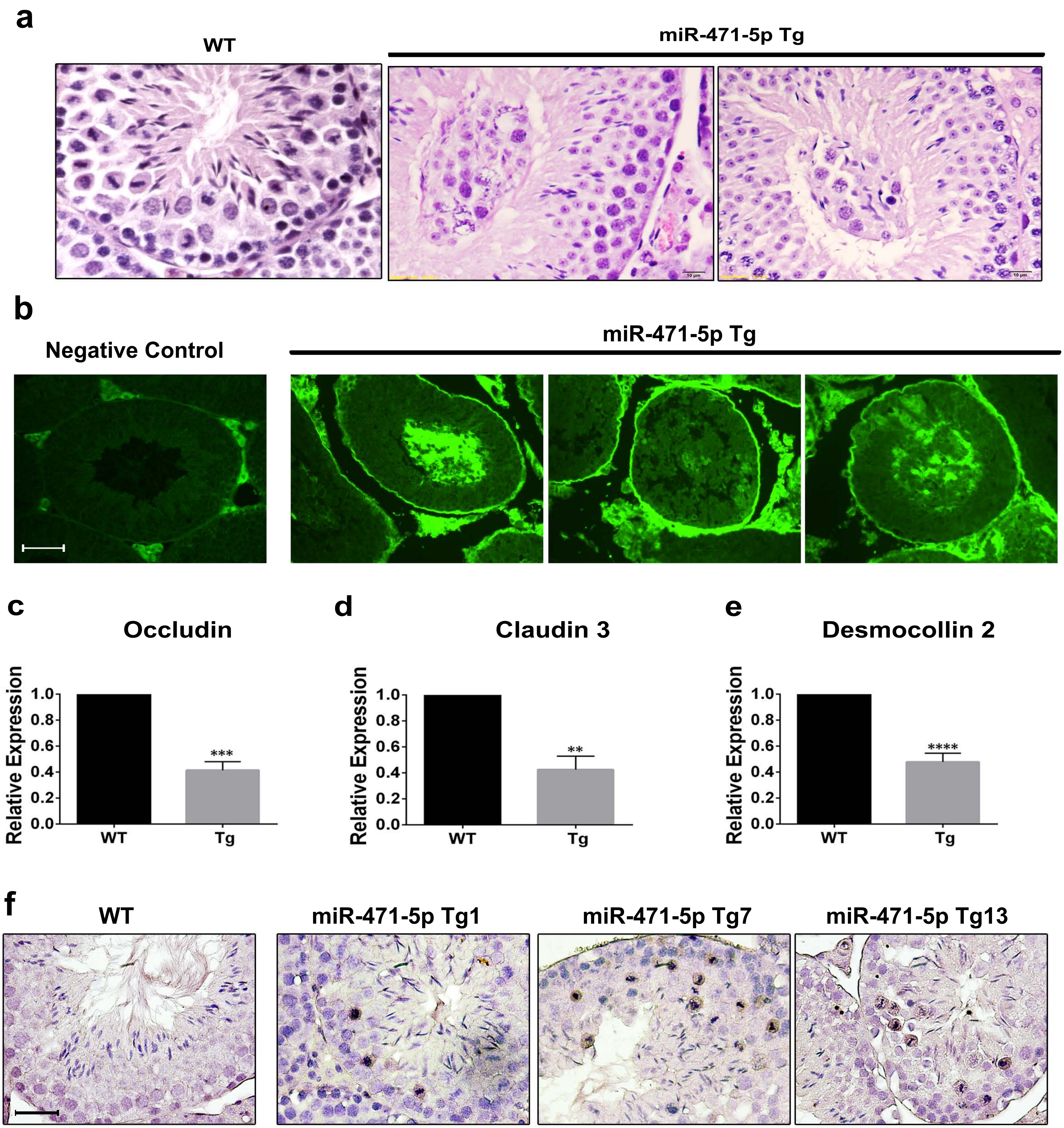

Supplementary Figure 2. Compromised blood–testis barrier integrity and increased apoptosis in miR-471-5p Tg mice. (a) Germ cells sloughing in the lumen of seminiferous tubules from wild type control (WT) and miR-471-5p Tg mice. (b) Immunofluorescence analysis showing biotin tracer dye in the adluminal compartment in miR-471-5p Tg mice. Negative antibody control shows no staining inside the seminiferous tubule. (c-e) RT-qPCR analysis showing reduced levels of adherens junction genes occludin (c), claudin3 (d) and Dsc2 (e) in the miR-471-5p Tg mice testis compared with the normal control (WT) testis. Data are presented as mean ± SEM of three independent experiments; ***$p < 0.001$, **$p < 0.05$; ****$p < 0.0001$, two tailed unpaired student t-test. (f) Immunohistochemical analysis (IHC) using antibody against cleaved caspase 3 showing germ cells undergoing apoptosis in miR-471-5p Tg lines. Scale bar indicates 10 μM (b), 20 μM (f).

Supplementary Figure 3

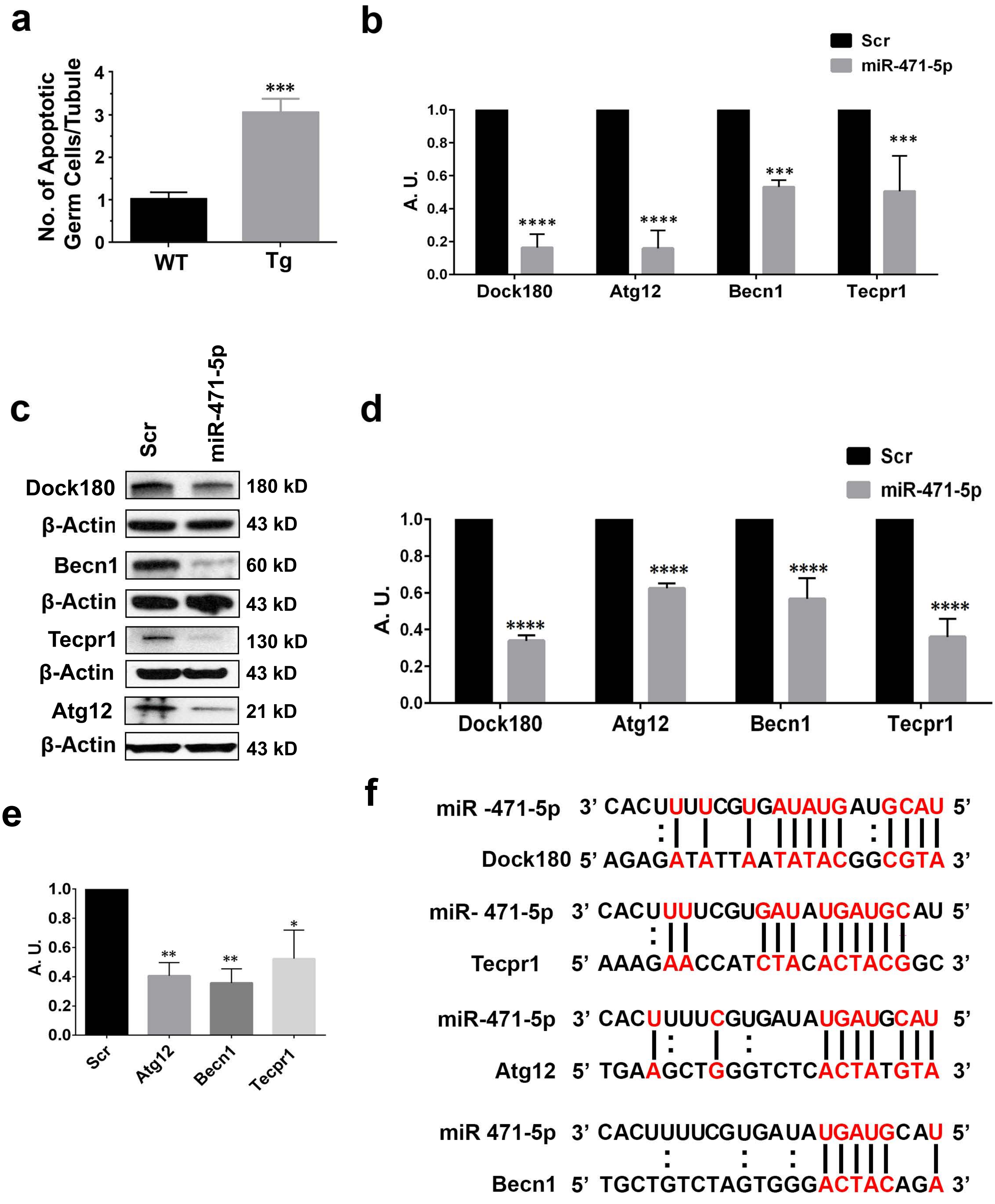

Supplementary Figure 3. Defective clearance of apoptotic germ cells in miR-471-5p Tg mice due to altered levels of autophagy-associated proteins. (a) TUNEL analysis of apoptotic germ cells in testes sections from three miR-471-5p transgenic lines showing average number of apoptotic germ cells per seminiferous tubules in WT and miR-471-5p Tg mice. Data are presented as mean ± SEM (WT: n=3 animals; 250 tubules/animal; miR-471-5p Tg: n=3 lines; 3 animals/line; 250 tubules/animal).***$p < 0.001$, two tailed unpaired student t-test. (b) Bar graph showing levels of autophagy-associated proteins in scramble or miR-471-5p-transfected 15p1 cells. Band intensities were quantified and normalized to β-actin from at least three independent experiments using ImageJ software. A representative gel photograph is shown in Fig. 2c. ***$p < 0.001$; ****$p < 0.0001$, two tailed unpaired student t-test. (c) Western blot analysis of TM4 cells transfected with scramble or *miR-471-5p* mimic using antibodies against indicated proteins. β-actin served as a loading control. Gel photograph is representative of three independent experiments. (d) Bar graph reflects band intensities (as described in c) quantified using ImageJ gel analysis software. ****$p < 0.0001$, two tailed unpaired student t-test. (e) Bar graph showing levels of indicated proteins in purified Sertoli cells isolated from WT and miR-471-5pTg. Band intensities were quantified and normalized to β-actin from atleast three independent experiments using ImageJ software. A representative gel photograph is shown in Fig. 2e. *$p < 0.05$; **$p < 0.01$, one-way ANOVA followed by Dunnett's multiple comparisons test.(f) Putative *miR-471-5p* binding sequence in the Dock180, Tecpr1, Atg12, and Becn1 3′ UTR.

Supplementary Figure 4

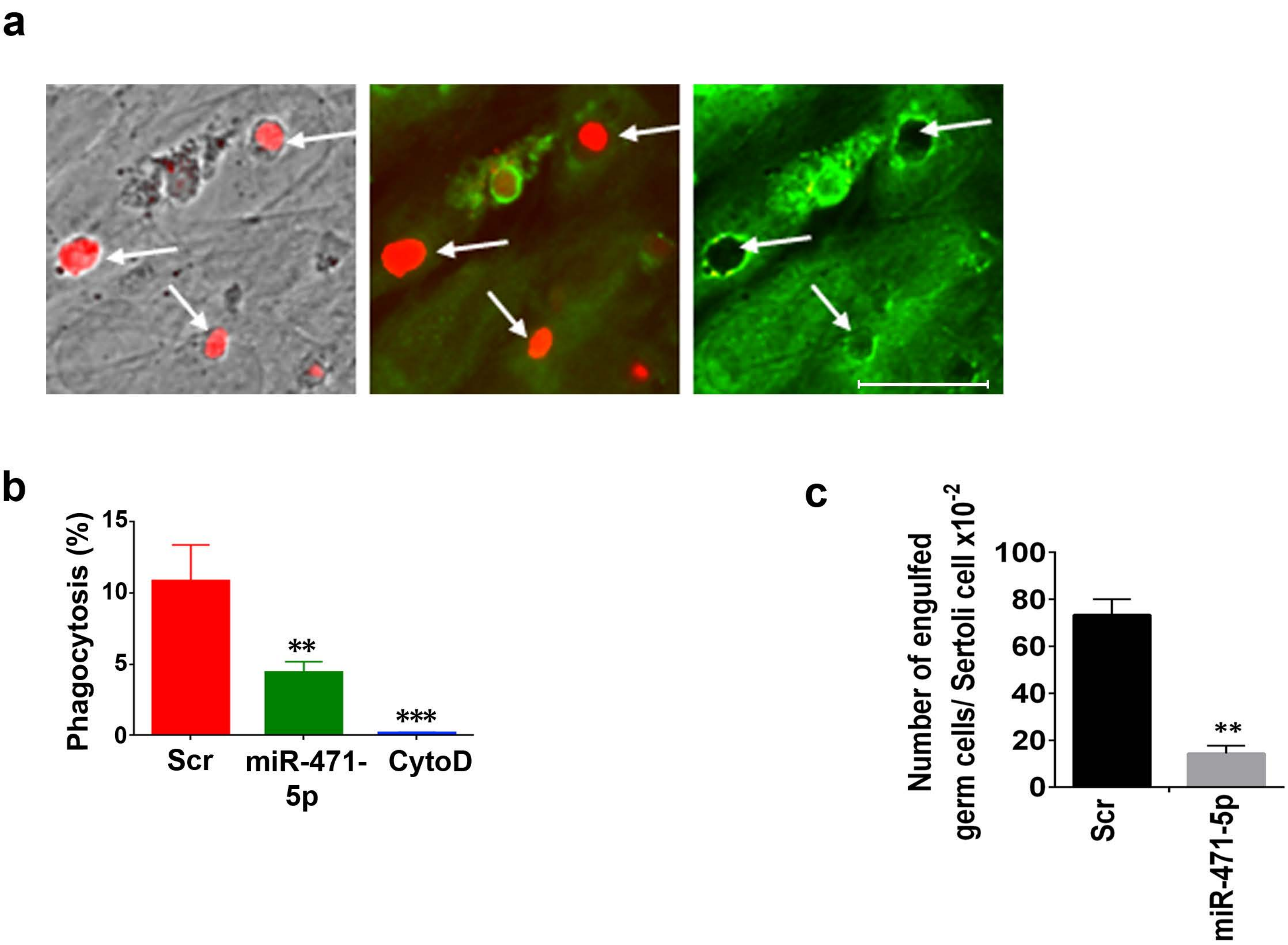

Supplementary Figure 4. MiR-471-5p regulates germ cell engulfment by the Sertoli cells. (a) Immunofluorescence analysis using propidium iodide labeled germ cells showing internalized apoptotic germ cells by the Sertoli cells. Engulfed germ cells are observed as phagocytotic cup. (b) Histogram showing percentage of control and miR-471-5p mimic (miR-471-5p)–transfected 15P1 Sertoli cells engulfing apoptotic germ cells as derived from the flow cytometric analysis. Cytochalasin D (CytoD), a phagocytosis inhibitor, used as a positive control. Data are presented as mean ± SEM of three independent experiments.**p = 0.0019; **p = 0.0024, one-way ANOVA followed by Dunnett's multiple comparisons test. (c) Histogram showing number of apoptotic germ cells engulfed per Sertoli cell in scramble (Scr) and miR-471-5p mimic (miR-471-5p) transfected primary Sertoli cells as derived from immunofluorescence analysis. Data are presented as mean ± SEM of three independent experiments (100 Sertoli cells/ group/experiment were counted). **P<0.01, two tailed unpaired student t-test. Scale bar indicates 10 µM (a).

Supplementary Figure 5

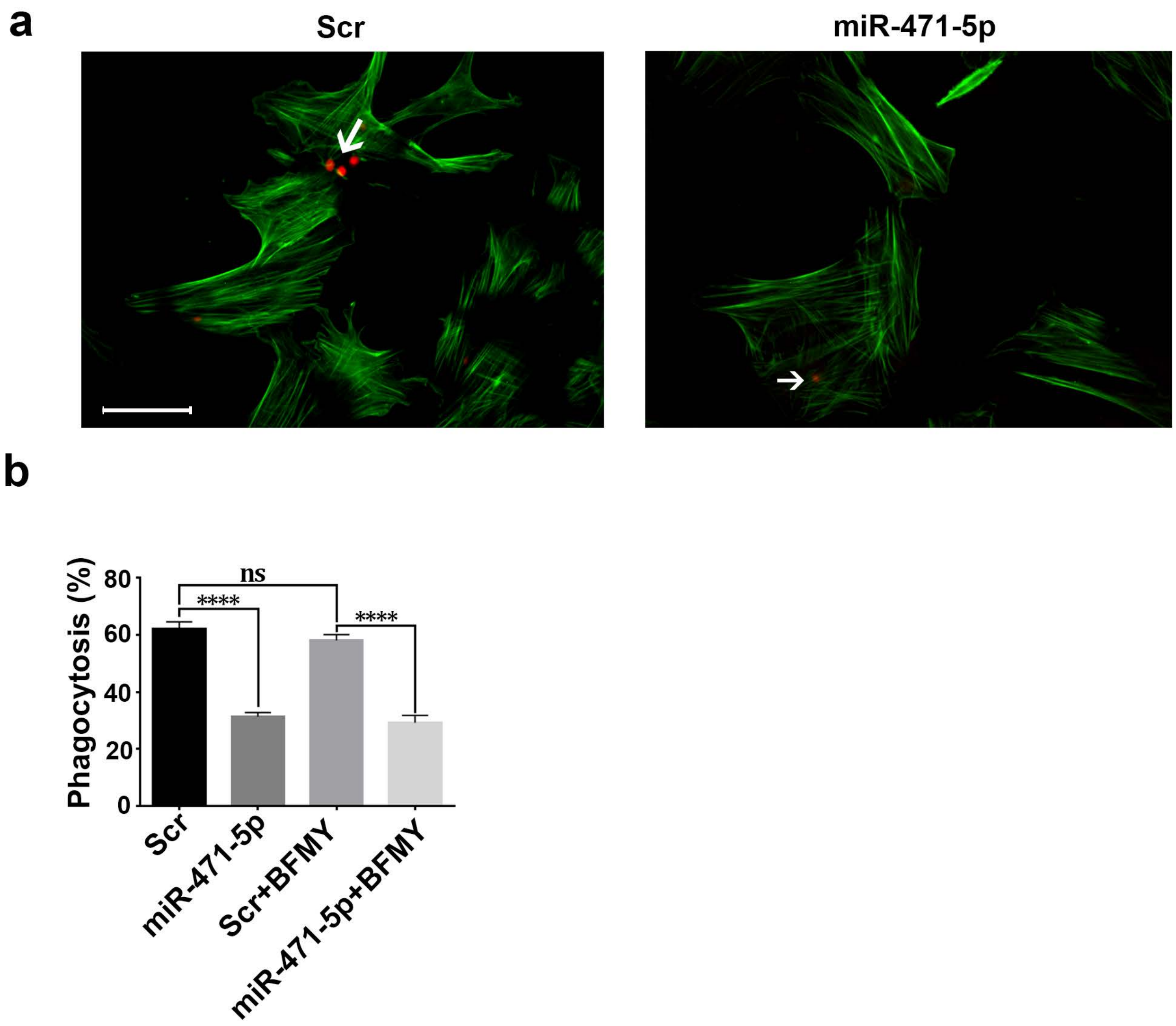

Supplementary Figure 5. MiR-471-5p regulates Sertoli cell phagocytosis. (a) Phagocytosis assay using pH-Rodo-labeled apoptotic germ cells in 15P1 Sertoli cells transfected with scramble or miR-471-5p mimic (miR-471-5p). (b) Histogram showing percentage of scramble or miR-471-5p mimic–transfected Sertoli cells engulfing apoptotic germ cells in the presence and absence of bafilomycin A1. Data are presented as mean ± SEM of three independent experiments (150 Sertoli cells were counted/experiment/group). ****$p < 0.0001$, two-way ANOVA followed by Tukey's multiple comparisons test. n.s. represents ‘not significant’. Scale bar indicates 10 µM (a).

Supplementary Figure 6

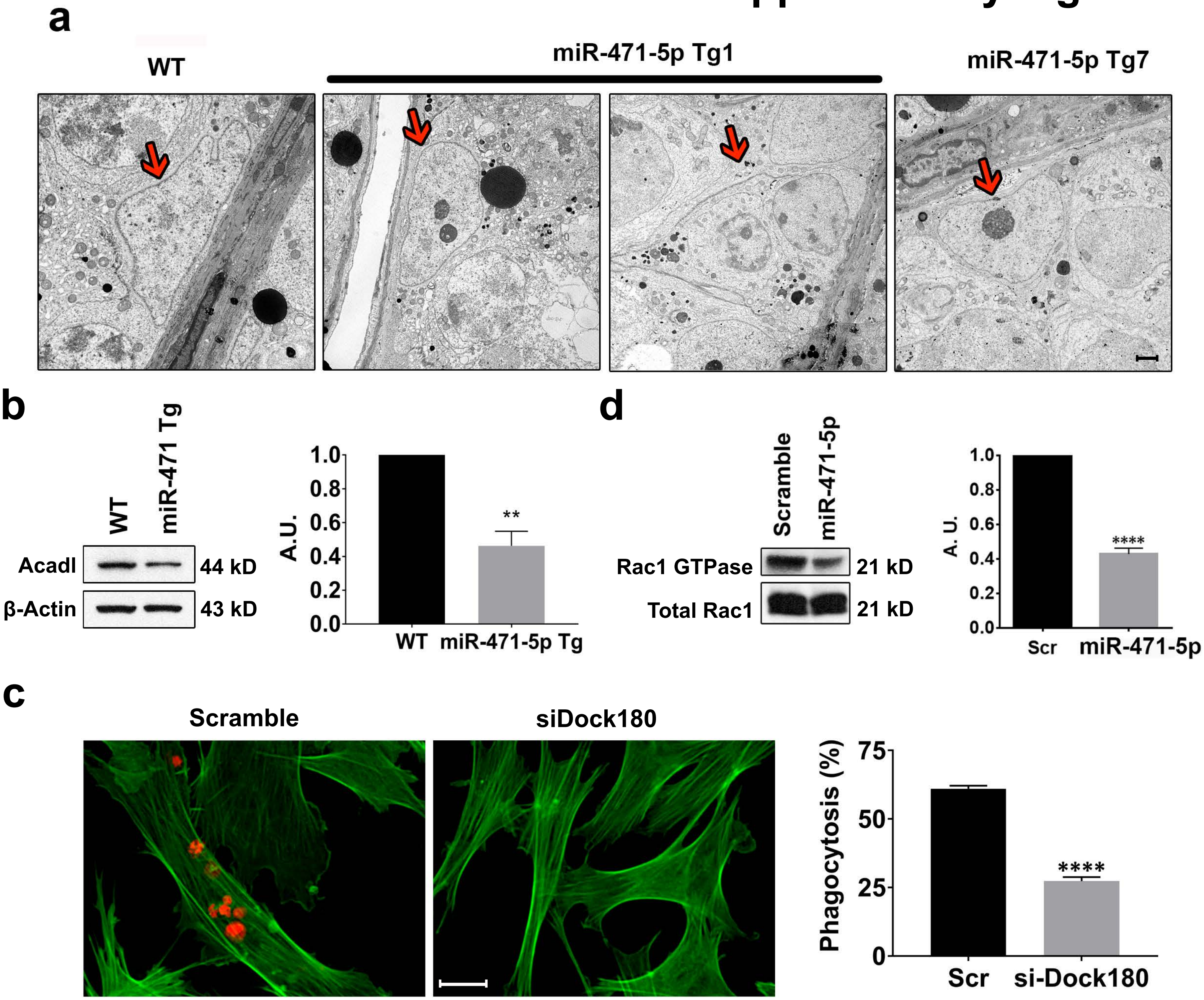

Supplementary Figure 6. MiR-471-5p regulates engulfment of apoptotic germ cells without affecting Sertoli cell morphology. (a) Ultrastructural examination of testicular sections from miR-471 Tg and normal control (WT) mice. (b) Western blot analysis on purified Sertoli cell lysates from WT and miR-471-5pTg mice (pooled from 3 mice/experiment; n=4) using antibodies against acyl-coenzyme A dehydrogenase, long-chain (ACADL). Bar graph shows band intensities quantified using ImageJ software. **P<0.01, two tailed unpaired student t-test. (c) Phagocytosis assay using pH-Rodo-labeled apoptotic germ cells in Sertoli cells transfected with scramble or si-Dock180. Histogram showing percentage of control and si-Dock180–transfected Sertoli cells engulfing apoptotic germ cells as derived from the immunofluorescence analysis. Data are presented as mean ± SEM of three independent experiments (100 Sertoli cells were counted/experiment/group).****p < 0.0001, two tailed unpaired student t-test. (d) Immunoprecipitation with active Rac1-GTPase followed by western blot analysis on scramble or miR-471-5p-transfected TM4 Sertoli cells using antibody against Rac1-GTPase. Input samples from both groups were used for western blot analysis using antibody against total RAC1. Gel photograph is representative of three independent experiments. Band intensities were quantified using ImageJ software. ****P<0.0001, two tailed unpaired student t-test. Scale bar indicates 2 μM (a), 10 μM (c).

Supplementary Figure 7

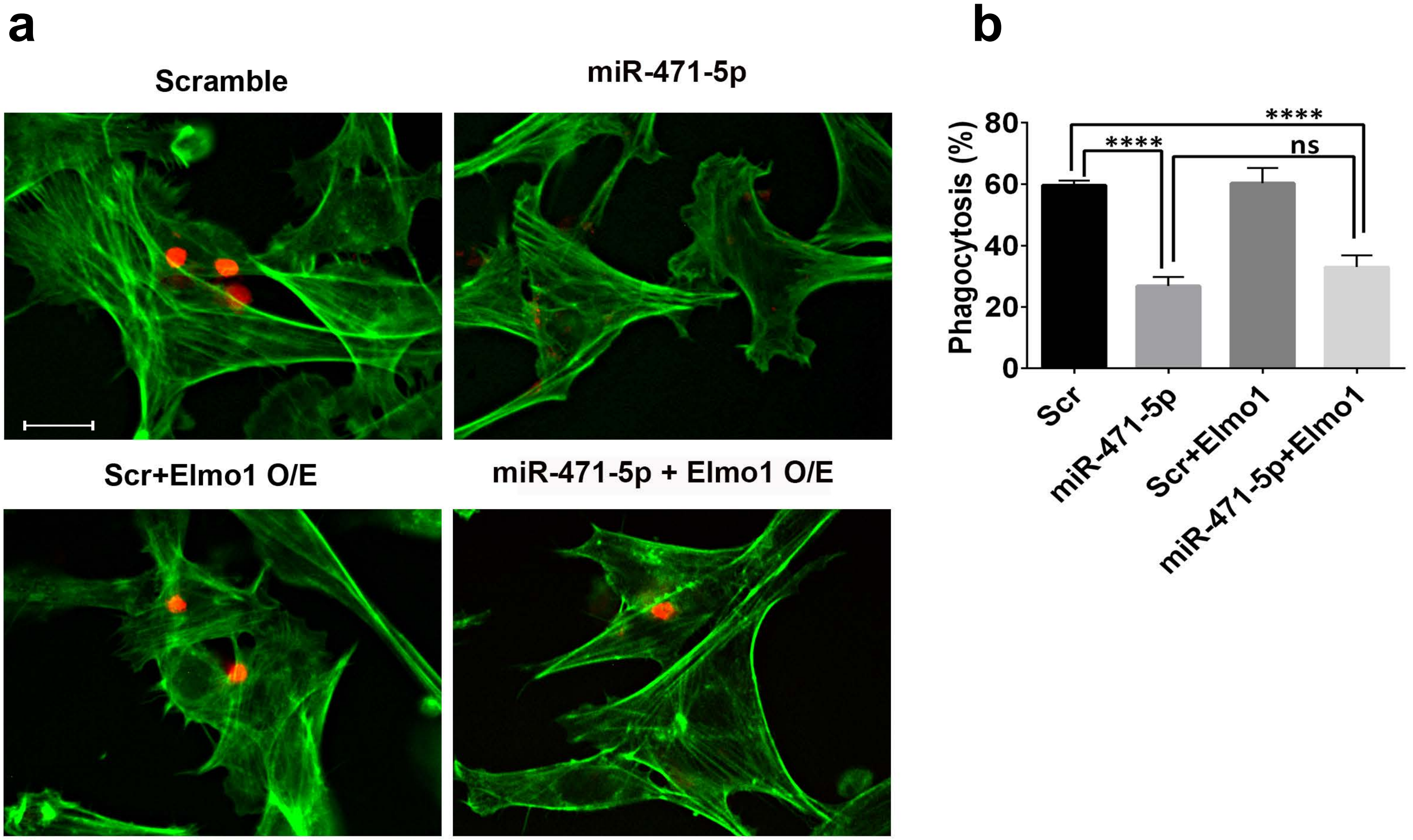

Supplementary Figure 7. miR-471-5p regulates phagocytosis (a) Immunofluorescence analysis showing engulfment of pH-Rodo-labeled apoptotic germ cells by TM4 Sertoli cells transfected with scramble, miR-471-5p mimic (miR-471-5p), Elmo1 expression construct or co-transfected with miR-471-5p mimic and Elmo1 expression construct. (b) Histogram showing number of Sertoli cells taking up apoptotic germ cells in experimental groups described in supplementary fig. 7(a). Data are presented as mean ± SEM of three independent experiments. ****$P<0.0001$, two-way ANOVA followed by Tukey's multiple comparisons test. Scale bar indicates 10 μM.

Supplementary Figure 8

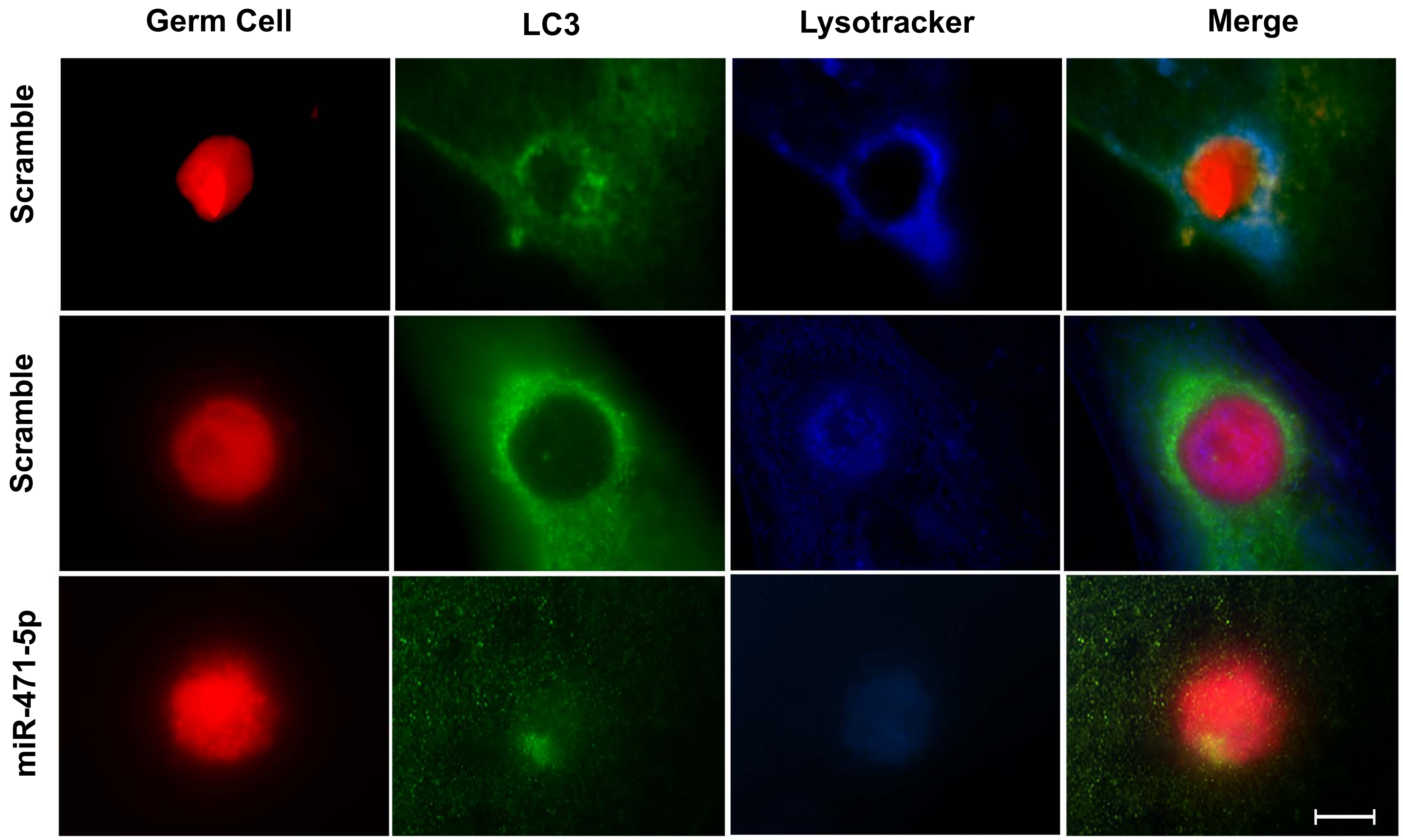

Supplementary Figure 8. miR-471-5p regulates LAP-mediated clearance of apoptotic germ cells. Immunofluorescence showing LC3 (green) and lysosomal (blue) coating around engulfed apoptotic germ cell (red). Primary Sertoli cells were transfected with scramble (first 2 rows) or miR-471-5p mimic (bottom panel) and preloaded with LysoTracker blue and fed with pH-Rodo-labeled apoptotic germ cells. Scale bar indicates 10 µM.

Supplementary Figure 9

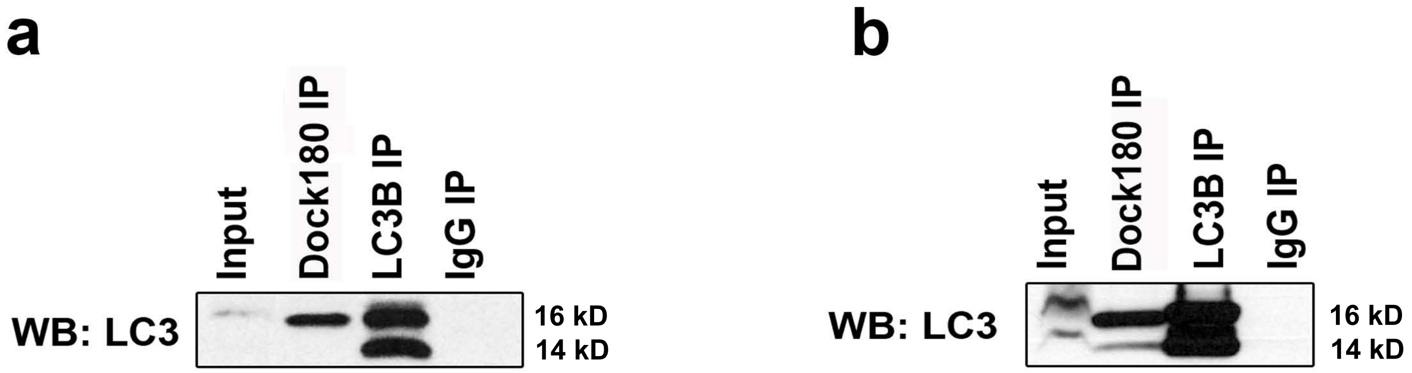

Supplementary Figure 9. Dock180 interacts with LC3B in mammalian cells. (a, b) Protein lysate from human mammary epithelial cells (HMEC) was immunoprecipitated with antibodies against Dock180, LC3B or IgG followed by western blot using antibody against LC3B. (b) Darker exposure showing LCBI and LC3BII bands. Gel photographs reflect representative of three independent experiments.

Supplementary Figure 10

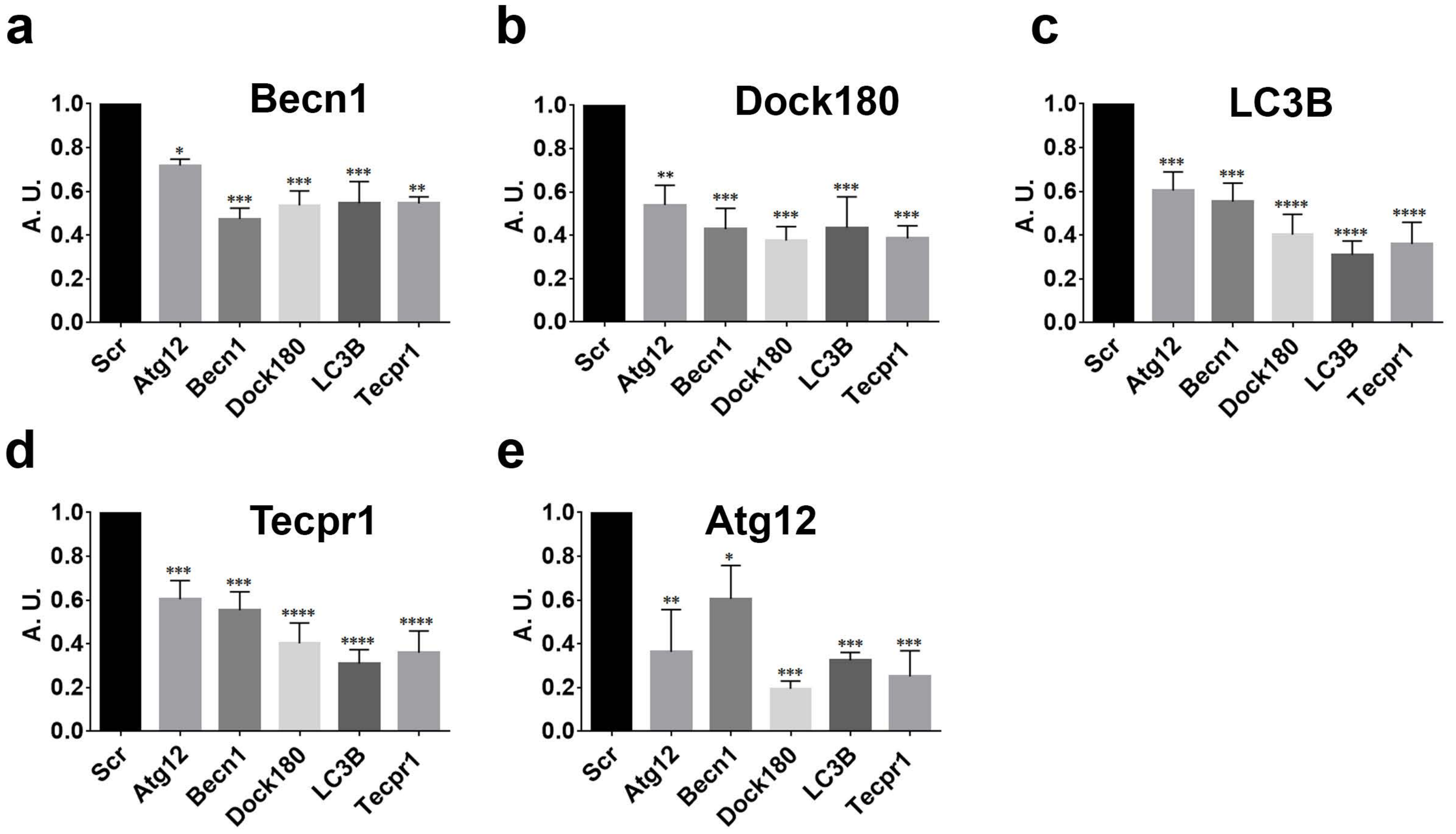

Supplementary Figure 10. Dock180, LC3B and autophagy associated proteins regulate each other's expression. (a-e) Bar graphs showing levels of indicated proteins obtained from western blot analysis on TM4 Sertoli cells transfected with siRNA against Scramble (Scr), Becn1 (a), Dock180 (b), LC3B (c), Tecpr1 (d) and Atg12 (e). Band intensities were quantified and normalized to β- actin from atleast three independent experiments using ImageJ software. A representative gel photograph is shown in Fig. 5f. *$p < 0.05$; **$p < 0.01$; ***$p < 0.001$; **** $p < 0.0001$, one-way ANOVA followed by Dunnett's multiple comparisons test.

Supplementary Figure 11

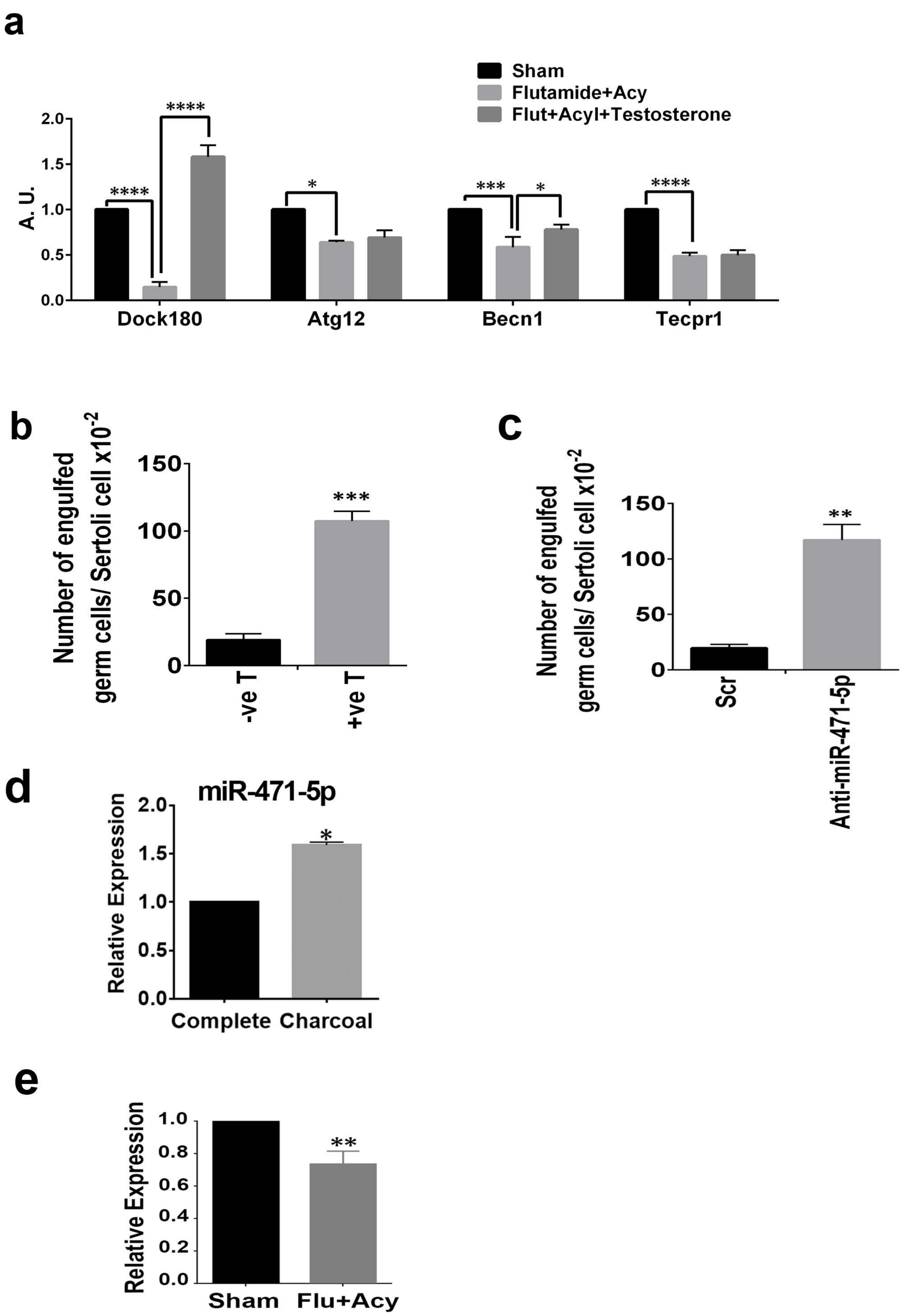

Supplementary Figure 11. Androgen regulates engufment of apoptotic germ cells by the Sertoli cells. (a) Bar graphs showing levels of Dock180, Atg12, Becn1, LC3B and Tecpr1 proteins obtained from western blot analysis (shown in Figure 6a) on Sertoli cells isolated from sham, flutamide + acyline (Flu+acy) and flutamide + acylene treated and supplemented with testosterone (Flu+acy+T) mice testes. Band intensities were quantified and normalized to β-actin using ImageJ software. A representative gel photograph is shown in Fig. 6a. *$p < 0.05$; **$p < 0.01$; **** $p < 0.0001$, two way ANOVA followed by Tukey's multiple comparisons test (b) Histogram showing number of germ cells engulfed/Sertoli cell in primary Sertoli cells isolated from normal control mice and cultured in the presence and absence of testosterone. Data are presented as mean ± SEM of three independent experiments. ***$p < 0.0002$, two tailed unpaired student t-test. (c) Histogram showing number of germ cells engulfed/Sertoli cell in scramble- and *miR-471-5p* antagomiR-transfected primary Sertoli cells isolated from flutamide-acyline treated mice and subsequently cultured in charcoal-stripped medium. Data in b and c are presented as mean ± SEM of three independent experiments (100 Sertoli cells were counted/group/experiment). **$p < 0.001$; two tailed unpaired student t-test. (d) qRT-PCR analysis showing *miR-471-5p* expression in primary Sertoli cells isolated from control and flutamide-treated mice and cultured in complete and charcoal stripped medium using *miR- 471-5p*-specific primers. Data are presented as mean ± SEM of three independent experiments. *$p < 0.05$, two tailed paired student t-test. (e) qRT-PCR analysis on RNA isolated from sham and flutamide–acyline-treated (Flu+Acy) mice testes using ACADL-specific primers. Data are presented as mean ± SEM of three independent experiments. **$p < 0.001$, two tailed unpaired student t-test.

Supplementary Figure 12

Fig. 2c

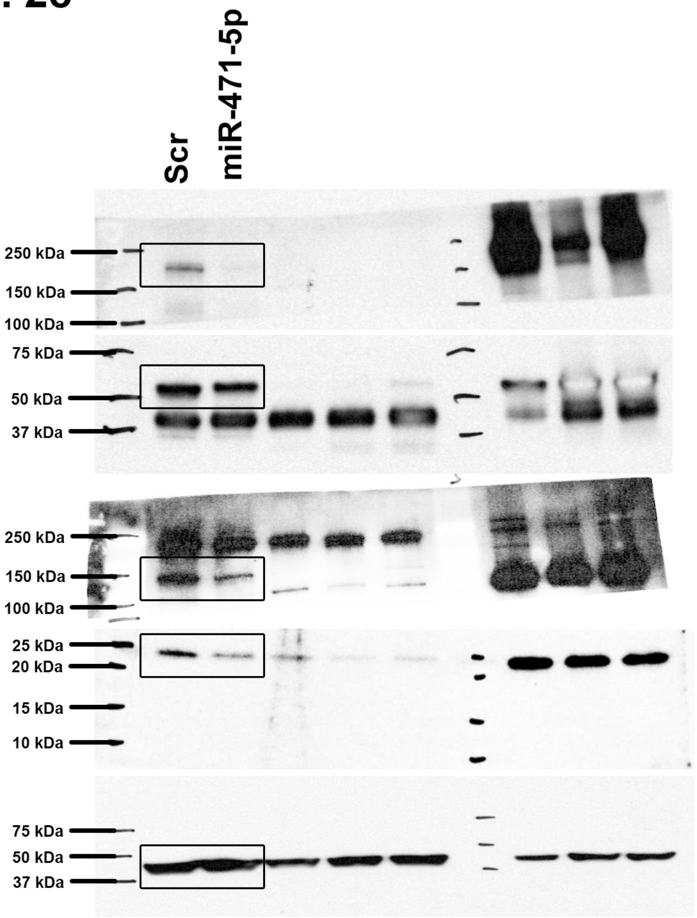

Fig. 2d

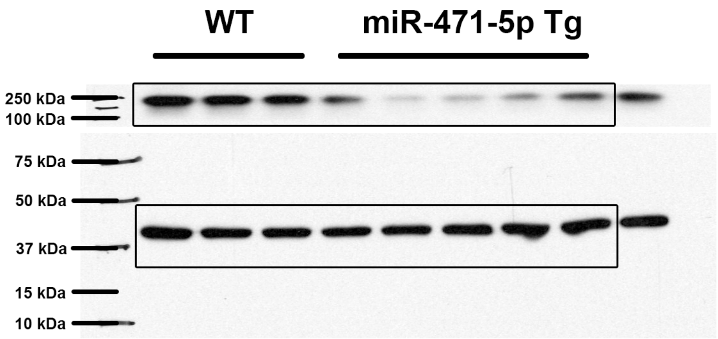

Fig. 2e

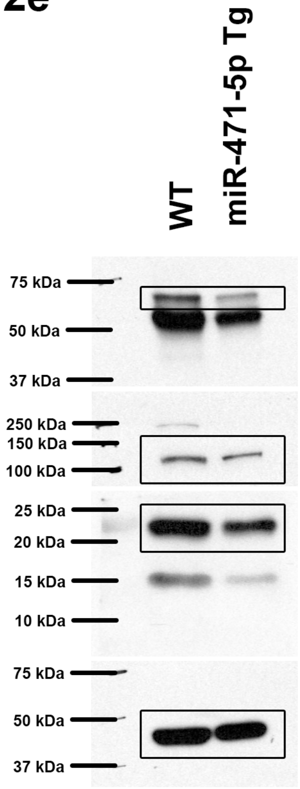

Figure 4a

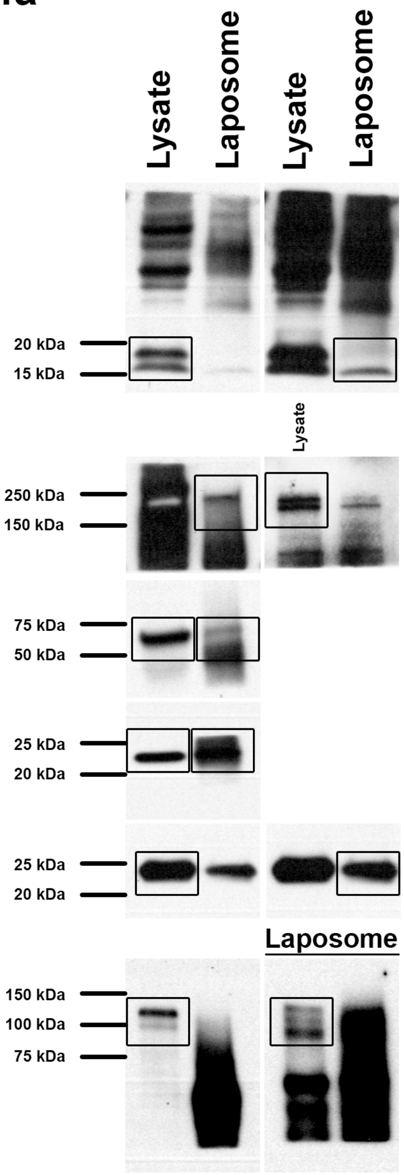

Supplementary Figure 12

Figure 4d

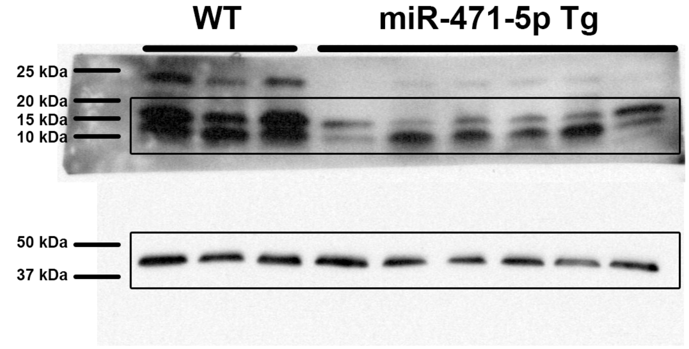

Figure 4e

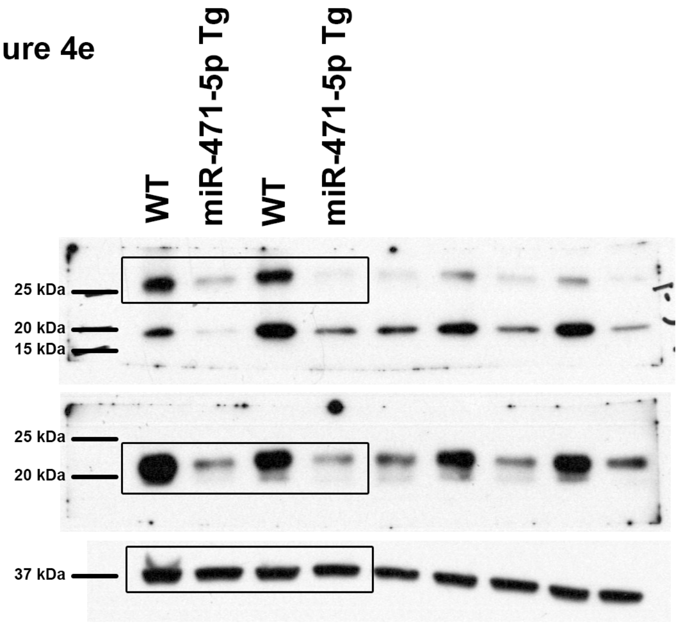

Figure 5b

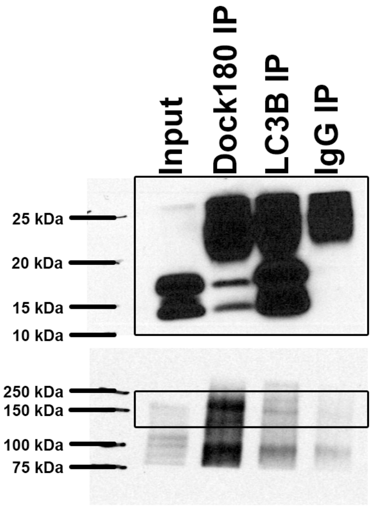

Figure 5c

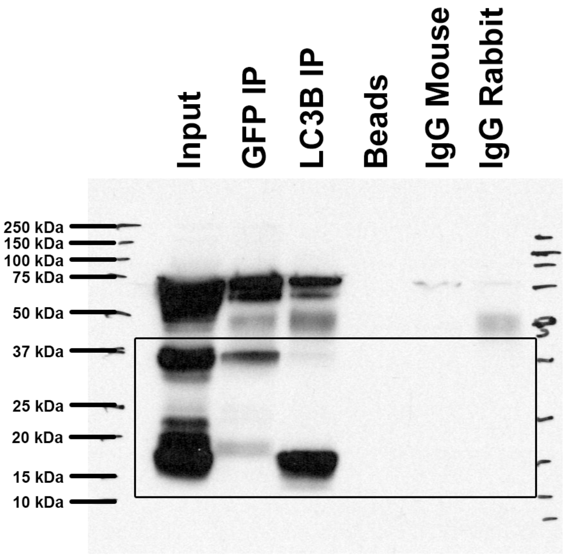

Figure 5e

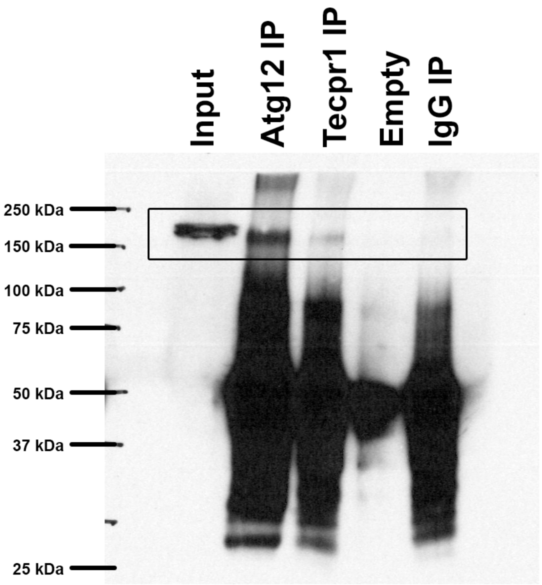

Figure 5d

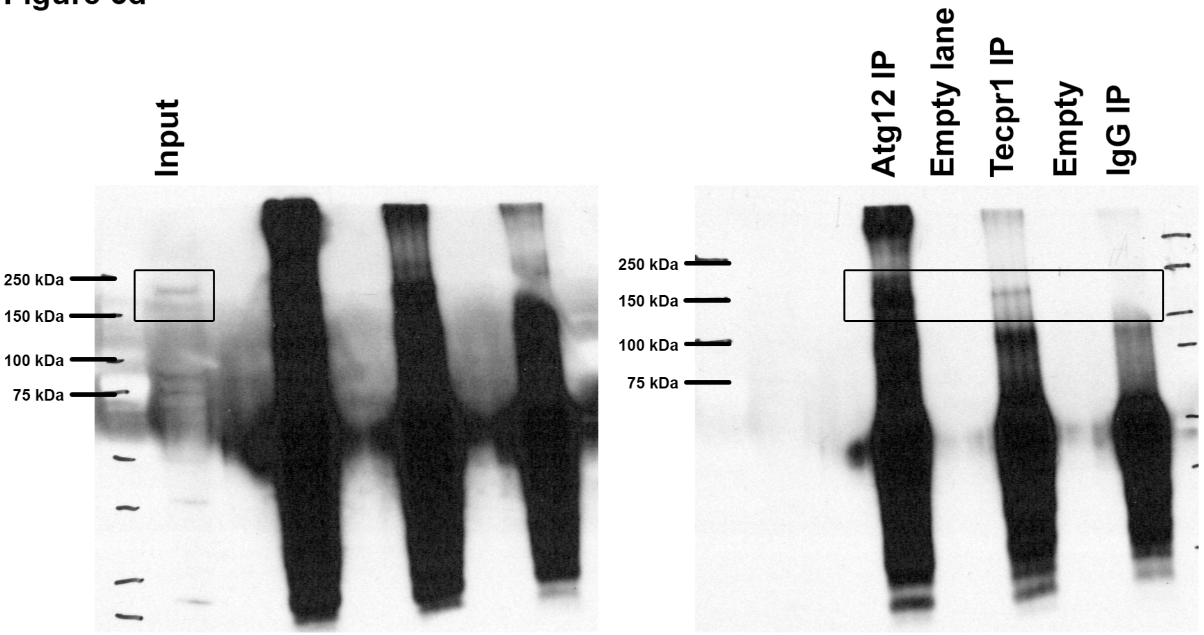

Supplementary Figure 12

Figure 5f

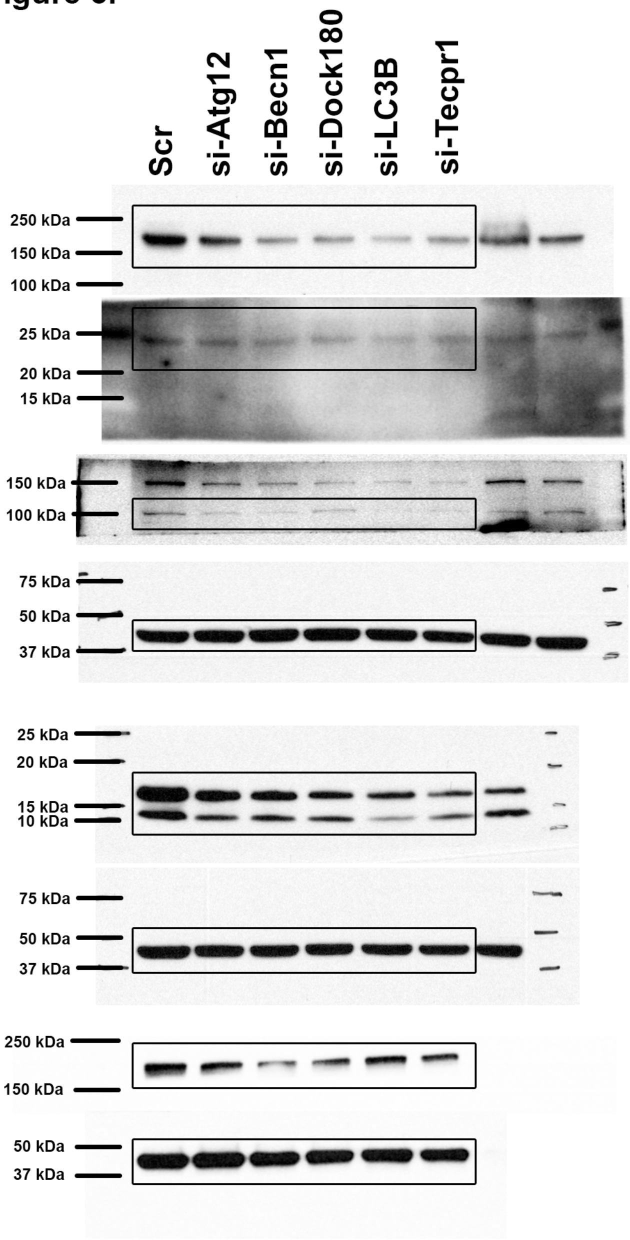

Figure 6a

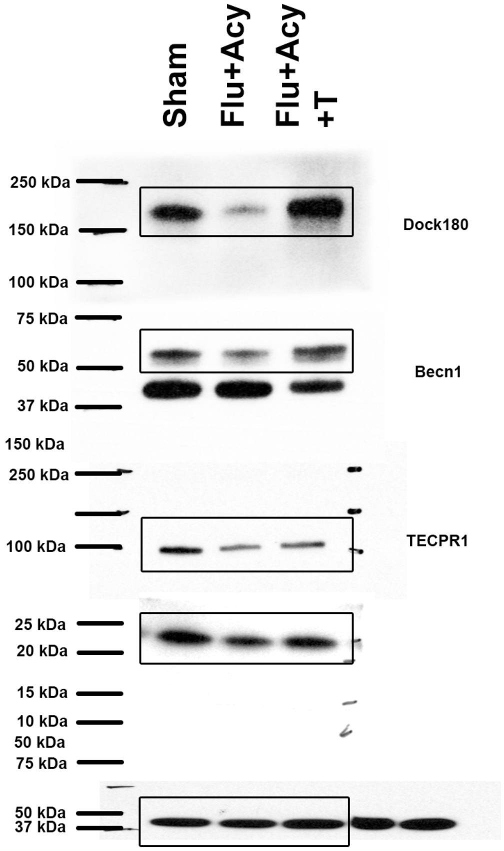

Supplementary Figure 12. Uncropped western blots. Cropped sections used as figures in the manuscript are marked as a box.

Supplementary Table 1: Differential expression of Autophagy/phagocytosis-related genes in *miR-471-5p* mimic transfected Sertoli cells compared with control as identified by Ago2-RNA RIP-Seq**.**

Gene	Fold change	P value
Fhl1	-6.24	0.0002
Acta2	-3.96	<0.0001
Tnfsf12	-2.85	0.0009
Pik3cd	-2.67	0.0176
Npy1r	-2.48	<0.0001
Zfp36	-2.39	<0.0001
Dusp1	-2.35	<0.0001
Rb1cc1	-1.82	0.0052
Pik3cb	-1.80	0.0391
Cnn2	-1.76	0.0082
Dock1	-1.75	0.0338
Elmo2	-1.66	0.0196
Ulk2	-1.55	0.0002
Dock4	-1.55	0.1232
Csk	-1.53	0.0018
Pik3c2b	-1.50	0.0249
Pik3c2a	-1.46	0.0309
Fyn	-1.45	0.0154
Dock7	-1.44	0.1043
Dock11	-1.44	0.0092
Tecpr1	-1.37	0.0005
Iqsec1	-1.35	0.003
Rab7	-1.35	0.0012
Mapk14	-1.33	0.062
Atg13	-1.33	0.0094
Atg2b	-1.28	0.1545
Atg12	-1.27	0.0058
Atg4d	-1.19	0.1135
Rab5a	-1.19	<0.0001
Ulk4	-1.17	0.0009
Atg14	-1.16	<0.0001
Mtor	-1.15	0.0141
Rala	1.23	0.0001
Atg10	1.88	0.037
Ereg	2.12	<0.0001
Nos2	2.29	<0.0001

Avil	2.32	<0.0001
Lcn2	2.36	<0.0001
Sspo	2.51	<0.0001
Dcn	4.36	<0.0001

Supplementary Table 2: Primers used in this investigation

Sl. No.	Gene	Primer sequence
1	BGH polyA (tail integration)	5′-CTGTGCCTTCTAGTTGCC-3′
		5′-GCCTGCTATTGTCTTCCC-3′
2	BGH polyA (qPCR)	5′-CTGTGCCTTCTAGTTGCC-3′
		5′-GGAAAGGACAGTGGGAG-3′
3	DOCK180	5′-CAGGAAGCATAAATACCTCGCC-3′
		5′-CAGCTCATCCGATTGTCTTTGT-3′
4	miR-471 cloning primer	5′-CAGAAGGTAAGATAGGAAGGACAT-3′
		5′-AATATCCCCTTGAGGAACAAC-3′
5	Ocln	5′- TTGAAAGTCCACCTCCTTACAGA-3′
		5′- CCGGATAAAAAGAGTACGCTGG-3′
6	Cldn3	5′- ACCAACTGCGTACAAGACGAG-3′
		5′- CAGAGCCGCCAACAGGAAA-3′
7	GAPDH	5′-AGGTCGGTGTGAACGGATTTG-3′
		5′-TGTAGACCATGTAGTTGAGGTCA-3′
8	ACTB	5′-GGCTGTATTCCCCTCCATCG-3′
		5′-CCAGTTGGTAACAATGCCATGT-3′
9	DOCK180 3′ UTR	5′-GGACTAGT GCTTGTCCAGTGTGAGTCAG -3′
		5′-GCGAGCTCCTGTACACACAGAGAAGCCACCC-3′
10	miR-471	5′-TAC GTA GTA TAG TGC TTT TCA-3′
11	RNU19	5′-TGT GGT GCC CGA GAT CGT-3′
		5′-TGG GAG CCG ACC CTT AGT AA-3′
12	5S rRNA	5′-AGG GTC GGG CCT GGT TAG TA-3′
		5′-CCT ACA GCA CCC GGT ATT CC-3′
13	ACADL	5′-TTT CCT CGG AGC ATG ACA TTT T-3′
		5′-GCC AGC TTT TTC CCA GAC CT-3′
